# Evaluation of Dimercaptosuccinic Acid-Coated Iron Nanoparticles Immunotargeted to Amyloid Beta as MRI Contrast Agents for the Diagnosis of Alzheimer’s Disease

**DOI:** 10.3390/cells12182279

**Published:** 2023-09-14

**Authors:** Marina Ulanova, Lucy Gloag, Andre Bongers, Chul-Kyu Kim, Hong Thien Kim Duong, Ha Na Kim, John Justin Gooding, Richard D. Tilley, Joanna Biazik, Wei Wen, Perminder S. Sachdev, Nady Braidy

**Affiliations:** 1 Centre for Healthy Brain Ageing, University of New South Wales, Sydney, NSW 2052, Australia; m.ulanova@unswalumni.com (M.U.); chulkyu.kim@unsw.edu.au (C.-K.K.); w.wen@unsw.edu.au (W.W.); p.sachdev@unsw.edu.au (P.S.S.); 2 Faculty of Science, School of Mathematical and Physical Science, University of Technology Sydney, Sydney, NSW 2007, Australia; lucy.gloag@uts.edu.au; 3 Mark Wainwright Analytical Centre, University of New South Wales, Sydney, NSW 2052, Australia; andre.bongers@unsw.edu.au (A.B.); r.tilley@unsw.edu.au (R.D.T.); joanna.richmond@unsw.edu.au (J.B.); 4 Faculty of Medicine, Prince of Wales Clinical School, University of New South Wales, Sydney, NSW 2052, Australia; 5 School of Chemistry, University of New South Wales, Sydney, NSW 2052, Australia; h.duong@unsw.edu.au (H.T.K.D.); justin.gooding@unsw.edu.au (J.J.G.); 6 Molecular Surface Interaction Laboratory, Mark Wainwright Analytical Centre, University of New South Wales, Sydney, NSW 2052, Australia; h.n.kim@unsw.edu.au; 7 Australian Centre for NanoMedicine, University of New South Wales, Sydney, NSW 2052, Australia; 8 Neuropsychiatric Institute, Euroa Centre, Prince of Wales Hospital, Sydney, NSW 2031, Australia

**Keywords:** Alzheimer’s disease, magnetic resonance imaging, iron nanoparticles, diagnosis

## Abstract

Nanoparticle-based magnetic contrast agents have opened the potential for magnetic resonance imaging (MRI) to be used for early non-invasive diagnosis of Alzheimer’s disease (AD). Accumulation of amyloid pathology in the brain has shown association with cognitive decline and tauopathy; hence, it is an effective biomarker for the early detection of AD. The aim of this study was to develop a biocompatible magnetic nanoparticle targeted to amyloid beta (A*β*) plaques to increase the sensitivity of T2-weighted MRI for imaging of amyloid pathology in AD. We presented novel iron core-iron oxide nanoparticles stabilized with a dimercaptosuccinic acid coating and functionalized with an anti-A*β* antibody. Nanoparticle biocompatibility and cellular internalization were evaluated in vitro in U-251 glioblastoma cells using cellular assays, proteomics, and transmission electron microscopy. Iron nanoparticles demonstrated no significant in vitro cytotoxicity, and electron microscopy results showed their movement through the endocytic cycle within the cell over a 24 h period. In addition, immunostaining and bio-layer interferometry confirmed the targeted nanoparticle’s binding affinity to amyloid species. The iron nanoparticles demonstrated favourable MRI contrast enhancement; however, the addition of the antibody resulted in a reduction in the relaxivity of the particles. The present work shows promising preliminary results in the development of a targeted non-invasive method of early AD diagnosis using contrast-enhanced MRI.

## 1. Introduction

Alzheimer’s disease (AD) is the most common neurodegenerative disease reported, contributing to 60–70% of the 50 million cases of dementia globally [1]. Owing to the multifactorial causes of dementia, a clinical diagnosis of AD primarily relies on ruling out other causes of cognitive decline. A definitive diagnosis of AD requires biomarker confirmation, which is currently only possible with a positron emission tomography (PET) scan or examination of the cerebrospinal fluid—each with limitations. AD is characterized by the deposition of amyloid beta (A*β*), neurofibrillary tau tangles, and neurodegeneration. The pathologic cascade of neuronal dysfunction, synaptic loss, and neuronal cell death that leads to cognitive decline in AD has been linked to the formation of neurotoxic A*β*_40–42_ oligomers and aggregated A*β* fibrils in plaques [2]. The presence of A*β* plaques has been demonstrated 10–20 years prior to the presentation of any clinical symptoms [3]; therefore, improved detection of A*β* plaques could enable AD diagnosis and treatment before significant neurological decline.

While cerebrospinal fluid A*β*_42_ concentration is considered a reliable biomarker used in AD diagnosis, the invasive nature of the lumbar puncture required to collect cerebrospinal fluid is a major limitation. Moreover, inconsistent interpretation of A*β*_42_ levels has been observed across clinical laboratories [4]. Imaging techniques, such as amyloid and tau PET, have facilitated improvements in the sensitivity of AD diagnosis. However, early-stage diagnosis and monitoring of the disease progression and response to treatment remains limited owing to the low spatial resolution of PET, the lack of widespread access to PET scanners, and the use of radioligands [5]. As an alternative diagnostic tool, magnetic resonance imaging (MRI) is more cost-effective and accessible, non-invasive, and can be rendered more sensitive with the addition of contrast agents. Gadolinium-based contrast agents, which are regularly employed in MRI imaging, have shown promise to delineate plaques both ex vivo and in vivo in animal models of AD [6,7,8,9,10]. Recent work using a liposomal macrocyclic Gd-DOTA contrast agent targeted with a DSPE-PEG3500-styryl-pyrimidine demonstrated 100% specificity at all dose levels using T1w-SE and FSE-IR, and a sensitivity greater than 80% at the highest dose level of 0.2 mmol Gd/kg. Interestingly, an optimal signal was observed 4 days following administration. However, gadolinium-based contrast agents are contraindicated for patients with renal impairment [11,12,13] and have been linked to the development of fatal cases of nephrogenic systemic fibrosis. Additionally, the deposition of gadolinium in the brain following administration of both linear [14] and macrocyclic [15] gadolinium contrast agents has been observed. Consequently, there is need for an alternative contrast enhancement formulation for the safe and sensitive diagnosis of AD. Other imaging tools, such as fluorine (^19^F) MRI, have been investigated for amyloid pathology detection [16,17,18]. ^19^F contrast agents are advantageous due to their high signal-to-noise ratio, as a lack of endogenous fluorine in soft tissue results in a negligible tissue background. However, efforts to develop clinically relevant probes are hindered by the need for large doses of ^19^F probes for adequate in vivo signals and current MRI hardware limitations.

Magnetic nanoparticles are formulated from biocompatible iron and can produce higher relaxation at lower doses. There are currently several United States Food and Drug Administration (FDA)-approved superparamagnetic iron oxide nanoformulations, which are used for liver, spleen, and gastrointestinal imaging [19]. Magnetic nanoparticles cause heterogeneity in the magnetic field, resulting in hypointense regions in T2-weighted MRI and improved relaxivity, and their high surface area permits functionalization with ligands targeted to specific pathologies. Various ligands, including fluorescent probes [20,21,22], A*β* peptides [23,24,25,26], antibodies [27,28,29,30,31,32], as well as hyaluronic acid [33], and lipocalin-type prostaglandin D2 synthase [34], have been employed to target AD pathology, showing promise as potential imaging agents for early diagnosis. However, research on these particles has not progressed past the preclinical stages. Nanoparticle interaction with and movement through the body is dynamic and complex, and is impacted by nanoparticle size, shape, and coating; therefore, a comprehensive characterization is essential for the development of a successful contrast agent.

Our current study adds to this emerging area of AD research by evaluating the in vitro MRI contrast enhancing efficacy and biocompatibility of dimercaptosuccinic acid (DMSA)-coated iron core-iron oxide shell nanoparticles functionalized with an antibody which binds A*β*. Iron core-iron oxide shell nanoparticles (hereafter referred to as iron nanoparticles) can achieve saturation magnetization values that are three times greater than similarly sized iron oxide nanoparticles, at sizes which are smaller than 20 nm, which is key to a strong MRI signal [35,36]. Furthermore, their small size is favourable for crossing the blood–brain barrier. DMSA-coated iron oxide nanoparticles have previously shown biocompatibility in numerous cell types, including HeLa cells [37], cultured primary neurons [38], skeletal myoblasts [39], and mesenchymal stem cells [40]. In the present study, we assessed the in vitro biocompatibility of DMSA-coated iron nanoparticles in U-251 glioblastoma cells, which are a commonly used astrocyte cell model and used in in vitro AD studies [41,42,43]. Astrocytes are abundantly distributed glial cells in the central nervous system and provide metabolic support to neurons. They comprise one of the cell layers of the blood–brain barrier [44], rendering them an important interface between the vasculature and the brain parenchyma. As such, it is vital to determine the biocompatibility of nanotechnology intended for use in the brain with this cell type. Previously, DMSA-coated iron oxide nanoparticles tested in cultured primary astrocytes [45] did not lead to acute cell death after treatment, with doses as high as 1000 µM of nanoparticles; however, these cells were treated for a maximum of 6 h. Furthermore, previous studies have shown that altered surface functionalization can change the biocompatibility of particles and influences the manner in which nanoparticles interact with the cellular environment. We therefore conducted comparative proteome analysis to evaluate proteomic changes in response to treatment with functionalized nanoparticles. Our results revealed that anti-A*β* conjugated iron nanoparticles can bind to A*β* plaques and enhance contrast in MRI in vitro. These non-toxic iron nanoparticles may be used for in vivo AD research that is non-invasive, non-toxic, and may ultimately lead to the realization of early AD diagnosis in humans. To our knowledge, this is the first study to evaluate the toxicology of targeted DMSA-coated magnetic nanoparticles in U-251 glioblastoma cells and provides an important contribution to the literature about the way in which different nanoparticle characteristics affect biocompatibility.

## 2. Materials and Methods

### 2.1. Synthesis of Iron Nanoparticles

Iron seeds were first synthesized, as described previously [35], by dissolving 0.3 g Fe(C_5_H_5_)(C_6_H_7_) in 6 mL of mesitylene (Sigma-Aldrich, St. Louis, MO, USA, purity: 98%) and 1.5 mL of oleylamine (70%, technical grade, Sigma-Aldrich, St. Louis, MO, USA). The reaction mixture was left to react in an oven at 130 °C for 72 h with 3 bar of hydrogen. The mixture was then allowed to cool to ambient temperature and transferred to an argon-filled glovebox. The iron seeds were subsequently re-reacted to grow the nanoparticles larger. Spherical nanoparticles were obtained by adding 1 mL of crude iron seed solution, a mixture of Fe(C_5_H_5_)(C_6_H_7_) iron precursor (0.1 g, 0.75 mmol), and 6 mL of mesitylene and oleylamine (0.5 mL, 0.75 mmol) and repeating the procedure 2 more times. The nanoparticles were precipitated through centrifugation at 4000 rpm for 30 min and redispersed in toluene (99.5%, Chem-Supply Pty Ltd., Gillman, SA, Australia) with a 100 mg/mL concentration [35].

The nanoparticles were coated with DMSA (Sigma-Aldrich, St. Louis, MO, USA, purity: ~98%) to allow dispersion in water. DMSA (20 mg) was dispersed in 1 mL of acetone and sonicated for 4 min. Then 2 mL of hexane and 2 mL of nanoparticle solution were added, gently shaken, and then mixed in a vortex mixer for 20 min. To the mixture, 25 µL of triethylamine (Sigma-Aldrich, St. Louis, MO, USA, purity ≥99.5%) was added and sonicated for 5 min. This step was repeated 3 more times to a total of 100 µL of triethylamine. Then, 2 mL of MilliQ water and 2–4 mL of hexane (95%, Chem-Supply Pty Ltd., Gillman, SA, Australia) were added to the mixture and left for 10 min to separate. The water layer was collected and purified 2× with ethanol (100%, Chem-supply Pty Ltd., Gillman, SA, Australia) via centrifugation at 4000 rpm for 10 min.

### 2.2. Particle Size and Zeta Potential

The hydrodynamic size distribution and zeta potential values of the iron nanoparticles dispersed in MilliQ water were measured using a dynamic light scattering (DLS) device from Malvern Instruments (Zetasizer Nano, Malvern, UK). Each sample was measured in triplicate at 25 °C after an equilibration step of 120 s using an acquisition time of 80 s. The hydrodynamic diameter was calculated using the DLS internal software.

Transmission electron microscopy (TEM) (Phillips CM200 field emission TEM) was used for the analysis of dry state size distribution and core–shell morphology. The software ImageJ was used for image analysis, with a minimum of 100 particles being counted for size distribution calculations.

### 2.3. Conjugation of Anti-Amyloid Antibodies to the Surface of Nanospheres

Rabbit anti-A*β*_1–42_ antibody (ab10148, Abcam, Cambridge, UK), was conjugated to the iron nanoparticles through carbodiimide reactions. An amount of 1 mg of DMSA-coated iron nanoparticles was resuspended in 0.1 M 2-(N-morpholino)methanesulfonic acid (MES) buffer at pH 6.2. To activate the carboxylic acids on the DMSA for conjugation with the antibodies, a 1-ethyl-3-(dimethylamino)propyl) carbodiimide (EDC) and sulfo-N-hydroxysuccinimide (NHS) (MES, EDC, and NHS: Sigma-Aldrich Pty Ltd., St. Louis, MO, USA) solution was prepared in MES buffer and added to the nanospheres at a final molar ratio of 1:5:10 (Fe:EDC:NHS). The particles were briefly sonicated and then mixed on a tube rotator at ambient temperature for 30 min. Iron nanoparticles were precipitated through centrifugation for 30 min at 3000 rpm. Activated iron nanoparticles were resuspended in 2 mL of 1x PBS containing 50 µg of anti-A*β* antibody. The iron nanoparticles were gently mixed by pipetting, and then the dispersion was mixed on a tube rotator for 2 h at 40 rpm at ambient temperature. The antibody-conjugated iron nanoparticles (referred to as NP-Ab) were washed by centrifugation at 3000 rpm for 30 min and redispersed in 1 mL of 1% BSA solution to block any unbound sites. This solution was mixed for a further 2 h on the tube rotator at ambient temperature. The NP-Ab were centrifuged for 30 min at 3000 rpm, redispersed in 1 mL of 1x PBS, and kept at 4 °C until use. Control iron nanoparticles, meaning those without conjugated antibodies, were resuspended in 1% BSA following EDC/NHS activation and incubated at 4 °C overnight. Control iron nanoparticles were subsequently washed by centrifugation for 30 min at 3000 rpm and resuspended in 1x PBS.

The successful conjugation of the antibody and quantification of the antibodies attached to the iron nanoparticles was confirmed using an enzyme-linked immunosorbent assay (ELISA). Briefly, 50 µL of NP-Ab and iron nanoparticle controls were incubated with a goat anti-rabbit IgG (HRP) secondary antibody (Abcam, Cambridge, UK, ab205718) for 45 min on a tube rotator. This was followed by washing three times in 1x PBS and using a magnet to retrieve the nanoparticles between washings. Iron nanoparticles and NP-Ab were resuspended in ELISA substrate and the colour change was observed. Furthermore, the binding of these NP-Ab to the A*β*_(1–42)_ peptide was characterized using BLItz/OCTET analysis. The method and results are described in detail in the Supplementary Methods (BLItz/OCTET Analysis for NP-Ab Binding to ProG Probe).

### 2.4. MRI of Nanoparticles

Iron nanoparticles were prepared for in vitro MRI by suspending iron nanoparticles and NP-Ab in a range of concentrations (0.1 µM, 0.2 µM, 0.5 µM, 1 µM, 100 µM, and 500 µM iron) in dH_2_O, with dH_2_O as a control. MR imaging for quantification of the T2 relaxation constants was performed at ambient temperature (22 °C) on a 9.4T Bruker (Karlsruhe, Germany) BioSpec Avance III 94/20 system equipped with a 72 mm internal diameter quadrature radiofrequency coil and BGA-12S HP gradients with a maximum strength of 660 mT/m and a slew rate of 4570 T/m/s. For imaging, all samples were positioned in an in-house, 3D-printed rack.

Image acquisition for T2 relaxation quantification was performed using a 2D Multi Spin Echo (MSE) pulse sequence. This protocol acquired an image series with 64 spin echo images in coronal orientation, covering a total echo time range from 10 ms to 662 ms. The following major sequence parameters were recorded: TR = 10,000 ms, first TE = 10 ms, DTE = 10.3 ms, 64 echoes, matrix size = 128 × 128, image in-plane resolution = 0.35 × 0.35 mm, slice thickness = 2 mm, Eff. spectral BW = 78,125 Hz, and total acquisition time with 2 ADC averages: 42 min.

T2 decay maps were calculated using pixelwise fittings of a mono exponential decay to the echo time image series.

From these maps, mean T2 relaxation times and 1/T2 relaxation rate (R2 in Hz) were calculated in ROIs covering the individual sample tubes. Nanoparticle relaxivity was then calculated through linear fittings of R2 vs. the sample concentration [mM].

### 2.5. Cell Culture

U-251 cells were obtained from the American Type Culture Collection (ATCC), sub-cultured, and then used to determine cytotoxicity after exposure to iron nanoparticles and NP-Ab. The cells were cultured in Gibco Roswell Park Memorial Institute (RPMI) 1640 media (Thermo Fisher Scientific), supplemented with 10% foetal bovine serum (FBS), 1% L-glutamine (Sigma-Aldrich, St. Louis, MO, USA), and 1% antibiotic/antimycotic (Sigma-Aldrich, St. Louis, MO, USA) at 37 °C under a humidified atmosphere of 5% CO_2_. At 85% confluency, the cells were harvested using trypsin/EDTA (0.25%) and phenol red (Gibco, Thermo Fisher Scientific) and seeded into T25 flasks, 24-well plates, or 96-well plates, according to the experiment being performed.

### 2.6. Lactate Dehydrogenase Assay

U-251 cells were used to evaluate the in vitro toxicity of iron nanoparticles and NP-Ab. Cells were seeded as described above into a flat-bottom 24-well plate and incubated at 37 °C. Iron nanoparticles were vortexed for 20 s and sonicated in a bath sonicator for 5 × 3 s with 5 s breaks in between to minimize agglomeration of the particles prior to cell treatment. NP-Ab were resuspended by gently pipetting. Different concentrations of both nanoparticles were added to the cells (0.1 µM−500 µM) and incubated for 24 h. Untreated cells were used as a negative blank control. All samples were assayed in triplicate. Following exposure, the cell supernatant was collected, and the cells were washed three times with 1x PBS. Then, 200 µL of 1x PBS was added to each well and a probe sonicator was used for 30 s in each well until the cells were completely homogenized.

Homogenized cells and supernatants were kept at −20 °C until analysis. A 200 µL aliquot of supernatant was used to perform a lactate dehydrogenase cytotoxicity assay (Thermo Fisher Scientific), normalized to protein level obtained with a protein BCA assay kit per the manufacturer’s instructions. Each sample was assayed in triplicate. One-way ANOVA with Tukey’s post hoc test was used to evaluate differences between treatment groups (GraphPad Prism, version 8.4.3).

### 2.7. Ames Bacterial Mutation Assay

Bacterial mutagenicity of iron nanoparticles was determined using the Muta-chromoplate™ Kit (EBPI, Burlington, ON, Canada), which is based on the widely used ‘Ames test’ [46]. This test uses a mutant strain of *Salmonella typhimurium* (TA100) carrying mutations in the operon coding for histidine biosynthesis and expressing the CYPP 1A1 enzyme. This test was carried out according to the manufacturer’s instructions. The growing *Salmonella typhimurium* TA100 strain culture was added to 96-well flat-bottomed plates, each containing a fixed concentration of iron nanoparticles. The plates were incubated at 37 °C for 3 days. Sodium azide was used as the positive control in this assay. The scores for the blank plate, the background control plates, and the positive control plates obtained were within the manufacturer’s specifications, indicating the validity of the results obtained for the aqueous extract.

### 2.8. Determination of Reactive Oxygen Species/Superoxide Production

The production of reactive oxygen species (ROS) and superoxide in cells in response to the iron nanoparticles was determined using a total ROS/Superoxide detection kit, according to the manufacturer’s instructions (Enzo Life Science, Farmingdale, NY, USA). Cells were seeded onto black-walled 96-well plates and incubated at 37 °C overnight in a CO_2_ incubator. The cells were treated with 3 concentrations of iron nanoparticles and incubated for 24 h. Each treatment had 6 replicates. The ROS inducer pyocyanin (200 µM) and 50 µL of MilliQ water were used as positive and negative controls, respectively. Following treatment, the solution was removed from each well and 100 µL of ROS/superoxide detection reagent was added. The plate was incubated for 30 min at 37 °C and measured in a fluorescence reader for fluorescein (for total ROS; excitation/emission = 488 nm/520 nm) and for rhodamine (for superoxide; excitation/emission = 550 nm/610 nm).

### 2.9. Quantification of Internalized Nanoparticles Using Inductively Coupled Plasma-Mass-Spectrometry

U-251 cells were incubated for 24 h with iron nanoparticles and NP-Ab with a total concentration of 5 µg of iron. Following incubation, supernatants were collected, and cells were washed three times with 1x PBS. Washes were pooled with supernatant to capture any nanoparticles that had detached from the cell surface. Wells were filled with 200 µL of 1x PBS and homogenized using a probe sonicator for 30 s. The iron concentration in the intracellular and extracellular compartments was evaluated using ICP-MS.

### 2.10. TEM Analysis of Nanoparticle Internalization into Cells

U-251 cells were grown on coverslips to 70% confluence and treated with nanoparticles for different incubation times (5 min, 1 h, 6 h, and 24 h), followed by fixation with 2% glutaraldehyde and 2% paraformaldehyde for 24 h at 4 °C. After fixation, the coverslips were washed in 0.1 M sodium phosphate buffer (pH 7.4), post-fixed in 2% OsO_4_ in 0.1 M sodium phosphate buffer for 60 min at 4 °C and dehydrated in increasing concentrations of ethanol. The samples were infiltrated with Procure 812 resin and polymerised at 60 °C for 48 h. Then, ultrathin (70 nm) sections were cut on a Leica EM UC6 Ultramicrotome and placed on carbon-coated copper mesh grids that were post-stained with uranyl acetate and lead citrate. Images were obtained using a Jeol JEM 1400 electron microscope operating at an accelerating voltage of 100 kV**.**

### 2.11. Immunohistochemistry

U-251 cells were grown on coverslips and incubated with NP-Ab and iron nanoparticles as for TEM imaging. Control cells were treated with 1x PBS at each time point. Following incubation, cell media was removed, and cells were fixed in 4% paraformaldehyde for 20 min at ambient temperature. The cells were washed three times with 1x PBS between each step. Antigen retrieval was performed in TE buffer (pH 9) at 90 °C for 10 min, followed by permeabilization with 0.25% Triton X-100 for 10 min. The cells were then incubated with a blocking buffer (comprising 0.5% normal goat serum, 1x PBS, and 0.1% Tween 20) for 40 min. Subsequently, the cells were incubated for 2 h at ambient temperature with a recombinant Alexa Fluor^®^ 488 rabbit monoclonal anti-LAMP1 antibody (Abcam, Cambridge, UK, ab281758, 1:50). Finally, cell nuclei were counterstained with 0.5 mg/mL DAPI for 40 min and following washing, coverslips were mounted onto slides. Stained cells were imaged using an Aperio XT slide scanner. Fluorescence and brightfield images were taken to capture the nanoparticles and antibody staining. Phalloidin was used as an actin skeleton marker for fluorescence quantification using CellProfiler (Broad Institute, MIT, Cambridge, MA, USA).

### 2.12. U-251 Cell Proteomics

#### 2.12.1. Cell Culture and Nanoparticle Treatment

Once the cells had reached 80–90% confluency in the T25 flasks, a 100 µM concentration of either iron nanoparticles or NP-Ab was added to the cell media and incubated for 24 h under normal cell growth conditions. Untreated flasks were used as controls. For proteome analysis, the media was removed and fresh unsupplemented media was added prior to harvesting the cells using a cell scraper. Cells were centrifuged for 5 min at 500× *g* at ambient temperature; subsequently, the media was removed, and the cell pellet was resuspended in 1x PBS to wash. This was repeated 3 times. Finally, cells were resuspended in 200 mL of 1x PBS and frozen at −80 °C until further analysis.

#### 2.12.2. Cell Lysis and Protein Digestion

Cells were resuspended in RIPA lysis buffer (Thermo Scientific, Sydney, Australia) containing protease inhibitor, 0.1 mM phenylmethylsulfonyl fluoride (Roche, Sydney, Australia). Cells were lysed using 5 × 5 to 10 s bursts of the probe sonicator, with chilling on ice conducted in between the sonication steps.

Cysteine disulphides in proteins were reduced in 2 mM tris (2-carboxyethyl)phosphine (TCEP) at 60 °C for 60 min, followed by cysteine alkylation in iodoacetamide for 10 min at ambient temperature. To remove detergents, reducing and alkylating agents and other non-protein components, and 1 mL of cold acetone containing 1% HCl, were added to each sample and placed in a −20 °C freezer for 12–14 h to precipitate proteins. The protein precipitate was centrifuged (13,000 rpm, 20 min, ambient temperature), and the supernatant was decanted and discarded. The pellets were air dried for 2–3 min and resuspended in lysis buffer (5% SDS, 100 mM ammonium bicarbonate, pH 7.5). Proteins were captured and desalted using S-Trap Mini spin traps (Protifi, Farmingdale, NY, USA), following the manufacturer instructions. On-trap digestion was performed by adding 1 µg of sequencing-grade trypsin (Promega, Sydney, Australia) in 50 mM ammonium bicarbonate (100 µL) to each sample, overnight at 37 °C. Peptides were recovered from the S-trap using sequential elutions with 100 µL, each containing 0.2% formic acid and 50% acetonitrile, followed by a 2 min centrifugation (4000 rpm, ambient temperature) at each step, and pooling all eluents. The final pooled elutions were dried under vacuum centrifugation (SpeedVac, Thermo Scientific, Sydney, Australia) and the desiccated pellet was resuspended in 50 µL of 1% formic acid containing 0.2% heptafluorobutyric acid. Prior to LC-MS/MS analysis, protein concentration was estimated using a micro-spectrophotometer (DeNovix, Wilmington, DE, USA) measuring absorbance at a wavelength of 280 nm, and quantification of mg/mL protein was determined using the extinction coefficient of albumin (BSA).

#### 2.12.3. Tandem Mass Spectrometry and Database Searching

Biological triplicates of all samples were analysed using a Q-Exactive Plus mass spectrometer (Thermo Electron, Bremen, Germany) connected to a nano-LC, Dionex UltiMate 3000 high-performance liquid chromatography (HPLC) system (Thermo Scientific, Waltham, MA, USA), equipped with an autosampler (Dionex, Amsterdam, The Netherlands). Mass spectrometer parameters for data-dependent acquisition (DDA) analysis, as well as nano-LC conditions, have previously been described [47]. Briefly, peptides (∼3 μg on-column) were initially captured onto a C18 cartridge (Acclaim PepMap 100, 5 μm 100 Å, Thermo Scientific Dionex, Waltham, MA, USA), then switching to a capillary column (25 cm length, 350 μm o.d., 75 μm i.d.) containing reverse-phase packing (C18, Reprosil-Pur, 1.9 μm, 200 Å, Dr. Maisch GmbH, Ammerbuch-Entringen, Germany). Peptide elutions involved a 60 min run time and a binary gradient of 0–45% buffer B at 200 nL/min, and the buffers used in this procedure were buffer A (H_2_O/CH_3_CN of 98:2, containing 0.1% formic acid) and buffer B (H_2_O/CH_3_CN of 20:80, containing 0.1% formic acid). The mass spectrometer settings included the following parameters: ion spray voltage 2000 V, capillary temperature 300 °C, positive ion mode, survey scan acquired (*m*/*z* 375–1750), and up to 10 multiply charged ions (charge state ≥ 2+) isolated for MS/MS fragmentation.

#### 2.12.4. Bioinformatics and Statistical Analyses

The raw data files obtained from the mass spectrometer were subjected to analysis using MaxQuant version 2.1.3.0 (downloaded from https://www.maxquant.org (accessed on 9 August 2023)). The Uniprot database was used for searching and quantification, employing the label-free quantification (LFQ) method with a minimum ratio count of 2 and a minimum number of neighbours of 3. Default settings were used for all other identification and quantification parameters. One replication of the iron nanoparticles was excluded from the analysis due to low protein counts. Perseus computational platform version 2.0.6.0 (downloaded from https://www.maxquant.org/perseus (accessed on 9 August 2023)) was used for subsequent statistical processing [48]. Filtering of raw intensity values was performed based on the “Only identified by site”, “Reverse”, and “Potential contaminant” columns to eliminate false-positive hits. The filtered values were log2-transformed and filtered further based on a minimum of 2 valid values in at least one group. The missing values were replaced using a normal distribution with a width of 0.3 and a down shift of 1.8. Differential expression analysis was carried out using a fold change cut-off of >1.2 or <0.83, with a *p*-value threshold of <0.05, and the Student’s *t*-test was used for calculating the *p*-value. Gene ontology (GO) classification was performed with the aid of the DAVID website (https://david.ncifcrf.gov (accessed on 9 August 2023)), and the results were visualized using GraphPad PRISM 9. Canonical pathway analysis was performed with the Ingenuity pathway analysis (IPA) software (Ingenuity^®^ systems, www.ingeuity.com (accessed on 9 August 2023). Lastly, the significantly upregulated and downregulated proteins were analysed using the STRING database (https://string-db.org/ (accessed on 9 August 2023)) to determine the protein networks.

### 2.13. Animals

Brain sections used for staining were obtained from the brains of APP/PS1 and wild type (WT) mice under procedures approved by the University of New South Wales Animal Ethics Committee (Ethics number: 19/56b) and which were performed in accordance with the Australian National Health and Medical Research Council guidelines for the care and use of animals for scientific purposes. Mice were housed at a constant temperature of 21 °C with a 12 h light/12 h dark cycle with ad libitum access to standard chow and water. Animals were euthanized with a lethal dose of pentobarbitone (Lethabarb^®^ (Virbac, Milperra, NSW, Australia)). Their brains were extracted and fixed in 4% paraformaldehyde solution for 3 days, followed by paraffin embedding for sectioning on a microtome. Sagittal sections were cut at a 5 μm thickness.

#### Immunohistochemical Characterization of NP-Ab

To determine the ex vivo A*β*-binding ability of the NP-Ab, slides with APP/PS1 and WT mouse brain sections were deparaffinized and rehydrated for immunohistochemical staining using the EnVision G/2 Doublestain System, Rabbit/Mouse (DAB+/Permanent Red, K5361, Agilent Dako, Santa Clara, CA, USA), as per the manufacturers’ instructions. Adjacent sections on the same slide were incubated with either NP-Ab, rabbit polyclonal anti-A*β* antibody (1:600) as a positive control, or 1x PBS as a negative control for 20 min, followed 3,3′diaminobenzidine (liquid DAB+, DAKO, Glostrup, Denmark) for 5 min. Following three washes in 1x PBS, the slides were mounted with mounting medium and visualized using fluorescence microscopy (Zeiss AXIO microscope, Oberkochen, Germany).

## 3. Results

### 3.1. Nanoparticle Characterization

Spherical (Figure S1A,C) iron core-iron oxide shell nanoparticles were synthesized, with DMSA used as the stabilizing coating. The dry diameter of the unconjugated iron nanoparticles was 11.6 nm ± 0.9 nm, while the hydrodynamic size in suspension in water was 107.6 nm (PDI = 0.141), with a negative zeta potential of −22.7 ± 6.26 mV (Table 1, Figure S1). Conjugation of antibodies to the iron nanoparticles increased the hydrodynamic size to 369.5 nm (PDI = 0.994), while the zeta potential remained negative: −31.5 ± 4.12 mV. This increase was likely due to the excess EDC and NHS coupling reagents used to attach the antibodies and the additional adsorption of water molecules to these hydrophilic molecules. No sedimentation was observed, indicating that little to no aggregation occurred. Greater aqueous stability of particles was achieved with a higher electrostatic repulsive force between the particles; therefore, a zeta value that is far from zero is typically associated with more colloidally stable particles than a zeta potential closer to zero. ELISA results determined a successful conjugation of the antibody and indirect quantification determined 0.37 µg of Ab/mg Fe, approximately 12.19 antibodies per nanoparticle. The single peak in the DLS is consistent with the antibodies attaching evenly around the nanoparticles to give a spherical morphology. Furthermore, A*β* plaques in ex vivo brain sections of mice were successfully immunostained using NP-Ab in the place of a primary antibody, indicating the presence of active binding sites on the nanoparticle-conjugated antibodies (Figure S2). BLItz/OCTET analysis was used to determine the binding affinity of the NP-Ab to the A*β*_(1–42)_ peptide and rule out possible effects of the conjugation on antibody binding ability (Figures S3 and S4). A*β*_(1–42)_ peptides were used as they are fibrillogenic, the main constituent of A*β* plaques deposited in the brain [49], and more prone to rapid aggregation into pathogenic oligomers than the A*β*_(1–40)_ form of the peptide [50]. The binding of the NP-Ab to ProG (Figure S4A) demonstrated the availability of Fc regions on the conjugated antibody, indicating that the conformation of the antibody has not been destroyed by the conjugation. In a subsequent analysis, following the binding of the NP-Ab to ProG, the BLItZ ProG biosensor with bound NP-Ab was dipped a second time into the running buffer containing the A*β*_(1–42)_ peptide to approximate the NP-Ab-binding affinity for A*β*. The association and dissociation curves for the interaction between ProG-NP-Ab and A*β* are shown in Supplementary Figure S4B. Iron nanoparticles coated with BSA did not show binding to ProG in the absence of anti-A*β* antibody functionalization.

### 3.2. Cell Viability

**Figure 1:**
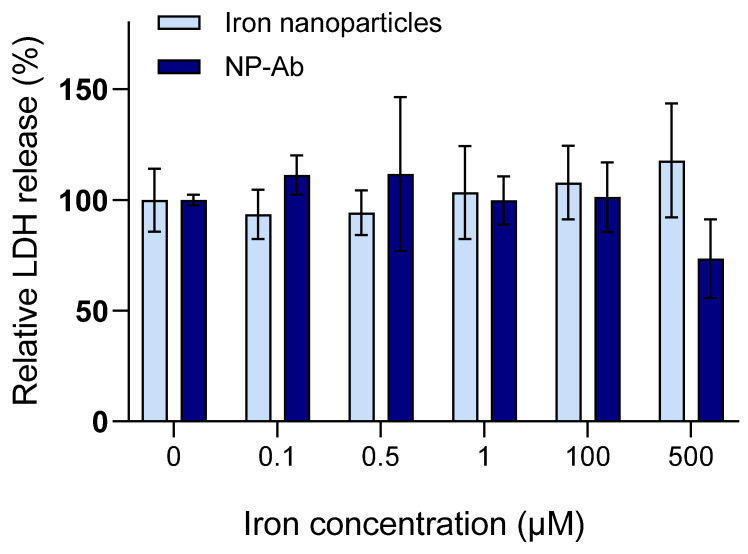
Iron nanoparticles and NP-Ab show biocompatibility. Lactate dehydrogenase release relative to untreated control wells in U-251 cells incubated with different concentrations of nanoparticles for 24 h. Data represent mean ± SD (*n* = 3–4). LDH: lactate dehydrogenase.

The cell viability following exposure to increasing concentrations of iron nanoparticles and NP-Ab was determined using a lactate dehydrogenase assay kit. Lactate dehydrogenase is released into the supernatant when the cellular plasma membrane is damaged and is a marker of cytotoxicity. A significant increase in lactate dehydrogenase release was not observed, even at the highest concentrations of 500 µM (Figure 1), in either the iron nanoparticle- or NP-Ab-treated cells. Interestingly, conjugated NP-Ab appeared to have a protective effect at the highest concentrations, resulting in a lower level of lactate dehydrogenase relative to the control.

### 3.3. ROS/Superoxide Production

Cells undergo oxidative stress as a protective mechanism against environmental stressors. Unconjugated iron nanoparticles were assessed and did not produce a significant increase in ROS production in U-251 cells, nor did they generate superoxide at any of the concentrations evaluated (Figure S5)

### 3.4. Mutagenicity of Iron Nanoparticles and NP-Ab in the Ames Test

Bacterial mutagenicity was assessed in vitro using the Ames kit in the *S. typhimurium* strain TA100. The bacterial strain was treated with 50 µM and 100 µM of iron nanoparticles and 50 µM of NP-Ab, and mutagenicity was determined based on the statistical table supplied by the manufacturer. Wells were considered positive if a colour change was observed. Some wells were observed to dry out during the incubation and were excluded from the total testable wells. The proportion of positive wells in the treated plates was compared to the results in the background plate, and results were classified as strongly mutagenic, moderately mutagenic, or mildly mutagenic if *p* < 0.001, <0.01, or <0.05, respectively. Iron nanoparticles only demonstrated mutagenicity at the higher 100 µM concentration. Conjugated NP-Ab did not show toxicity at 50 µM (Table S1).

### 3.5. Cellular Internalization of Iron Nanoparticles and NP-Ab

**Figure 2:**
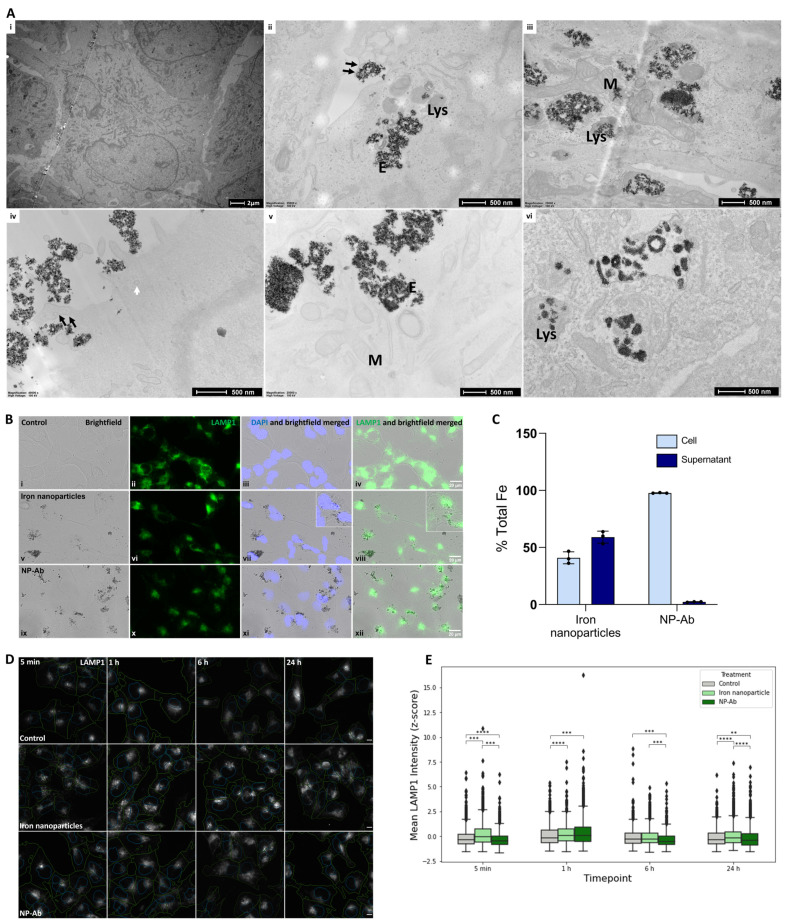
Iron nanoparticle and NP-Ab internalization and localization within U-251 cells. (**A**) Electron microscopy study of nanoparticle interaction and uptake by U-251 cells. Images from sections of U-251 cells incubated with iron nanoparticles (**i**–**iii**) and NP-Ab (**iv**–**vi**) for 5 min (**i**,**iv**), 6 h (**ii**,**v**), and 24 h (**iii**,**vi**). At 5 min, iron nanoparticles were not yet observed on the periphery of the cell (**i**). NP-Ab (**iv**) were primarily observed on the outside of the cell and in close proximity with the cell membrane. Membrane elongation around the iron nanoparticle (**ii**) and NP-Ab (**iv**) aggregates (>100 nm) suggests macropinocytosis as a potential internalization mechanism (double arrow). At 24 h (**iii**,**vi**), iron nanoparticles and NP-Ab were confined to the endosomes (E) and lysosomes (Lys). None were observed within mitochondria (M) (**B**) Brightfield, fluorescence, and their merged images for U-251 cells stained with LAMP1 (lysosomes, green) and DAPI (nuclei, blue) following incubation with 1xPBS (**i**–**iv**) iron nanoparticles (**v**–**vii**) and NP-Ab (**viii**–**xii**) for 24 h. ImageJ merging of the brightfield image over the fluorescent micrographs shows nanoparticle localisation within cells relative to the nuclei and lysosomes. (**C**) Quantitative cellular uptake by U-251 cells measured using ICP-MS after incubation with 5 µg Fe of iron nanoparticles or NP-Ab for 24 h. Data represent mean ± SD (*n* = 3). (**D**) Expression of LAMP1 markers (green) in U-251 cells treated with 1x PBS (control), iron nanoparticles, and NP-Ab at different timepoints. Cell nuclei and cytoplasm outlines are shown in cyan and green, respectively. Scale bars: 20 μm. (**E**) Quantification of single-cell mean fluorescence intensities. Total cells per treatment condition: control 24 h = 631, iron nanoparticle 24 h = 1938, NP-Ab 24 h = 2208, control 6 h = 899, iron nanoparticle 6 h = 1461, NP-Ab 6 h = 1148, control 1 h = 2606, iron nanoparticle 1 h = 960, NP-Ab 1 h = 2752, control 5 min = 2112, iron nanoparticle = 2500, and NP-Ab 5 min = 1860. Two-sided Mann–Whitney–Wilcoxon test, ns: (5.00 × 10^−2^ < *p* ≤ 1.00 × 10^0^), (1.00 × 10^−2^ < *p* ≤ 5.00 × 10^−2^), **: (1.00 × 10^−3^ < *p* ≤ 1.00 × 10^−2^), ***: (1.00 × 10^−4^ < *p* ≤ 1.00 × 10^−3^), and ****: (*p* ≤ 1.00 × 10^−4^).

ICP-MS and TEM analyses were conducted to determine the uptake and localization of iron nanoparticles and NP-Ab by U-251 cells. Following 24 h of incubation, approximately 95% of functionalized NP-Ab were internalized, while less than 50% of the non-functionalized iron nanoparticles were within the cells after that time (Figure 2C). The major advantage of this technique is that the nanoparticles do not need to be modified in any way, which can influence the internalization efficiency. For the TEM experiments, cells were exposed to 100 µM of iron nanoparticles and NP-Ab for 5 min, 6 h, and 24 h. Iron nanoparticles and NP-Ab both showed similar patterns of uptake and cellular localization (Figure 2A). Membrane protrusions were seen extending around > 100 nm agglomerates of iron nanoparticles (Figure 2A(ii), double arrows) and NP-Ab (Figure 2A(iv), double arrows), suggesting macropinocytosis as the uptake mechanism. Additionally, coated vesicles were visible alongside the periphery of the cells devoid of iron nanoparticles (Figure 2A(iv)), suggesting a clathrin-independent route. In the time between 6 h–24 h (Figure 2A(ii,iii,v,vi)) following incubation, iron nanoparticles and NP-Ab were localized within endosomes and lysosomes, suggesting the involvement of a degradation pathway or lysosomal exocytosis. Endosomes are apparent by their irregular and distended morphology encapsulating nanoparticles (Figure 2A(ii,v)), compared to the more spherical and electron-dense nanoparticle-containing lysosomes. Iron nanoparticles and NP-Ab did not induce morphological alterations in the cells and were not observed in the mitochondria, nuclei, or endoplasmic reticulum, nor were they freely dispersed in the cytoplasm. To investigate the activity of lysosomes in response to the iron nanoparticle and NP-Ab treatments, cells were incubated with iron nanoparticles, NP-Ab, or 1x PBS for the same time points as the TEM experiment (5 min, 1 h, 6 h, or 24 h) and stained for LAMP1. LAMP1 fluorescence intensity was then quantified using CellProfiler software. Merged micrographs of the brightfield with fluorescent images showed the colocation of the nanoparticles with lysosome staining and around the perinuclear region (Figure 2B). Some nanoparticles were observed over nuclear staining.

Compared to the control cells treated with 1x PBS, cells treated with iron nanoparticles showed a significantly higher LAMP1 signal intensity at all time points examined (*p* < 0.05) (Figure 2E). In contrast, NP-Ab-treated cells showed significantly lower LAMP1 intensity levels compared to the control cells at all time points except for 1 h (*p* < 0.05). Interestingly, the highest LAMP1 intensity was observed at 1 h following incubation in both the iron nanoparticle- and NP-Ab-treated cells. The exception observed at the 1 h time point may suggest a potential temporal effect of the treatment on LAMP1 expression, resulting in increased lysosomal activity. Meanwhile, at the later timepoints, the iron deposits, which can be observed in the brightfield images, may quench the fluorescent signal in the images.

### 3.6. MRI of Iron Nanoparticles and NP-Ab

**Figure 3:**
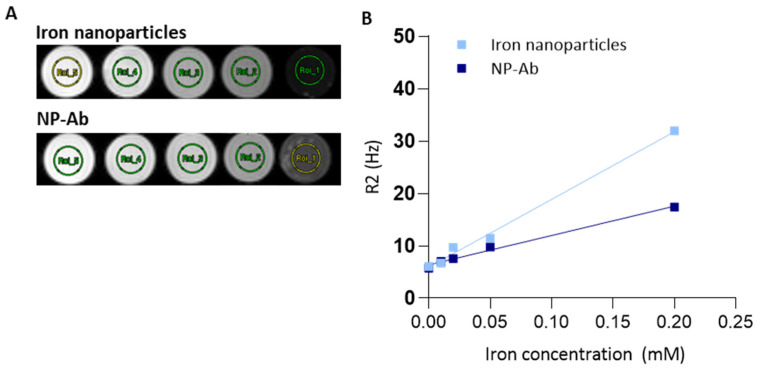
In vitro MRI properties and relaxometry of iron nanoparticles and NP-Ab. (**A**) MR imaging of iron nanoparticles and NP-Ab phantoms in MilliQ at increasing concentrations from left to right (0 mM, 0.01 mM, 0.02 mM, 0.05 mM, and 0.2 mM Fe). (**B**) Corresponding curves of the inverse of T2 (R2) versus concentration, where the gradient gives the *r*_2_ relaxivity in mM Fe^−1^, s^−1^.

To evaluate the contrast-enhancing ability of iron nanoparticles and conjugated NP-Ab, the transverse T2 relaxation times at 9.4 T were evaluated using in vitro MRI. Increasing the concentrations of both the iron nanoparticles and NP-Ab led to the concentration-dependent darkening of the MR images (Figure 3). Functionalized NP-Ab showed lower signal intensity levels than iron nanoparticles*,* with the relaxivity values of the iron nanoparticles and NP-Ab being 129.29 mM^−1^s^−1^ and 55.99 mM^−1^s^−1^, respectively. R2 measurements of the commercially available contrast agent Resovist were also completed (*r_2_ =* 183.88 mM^−1^s^−1^).

### 3.7. Proteomics

**Figure 4:**
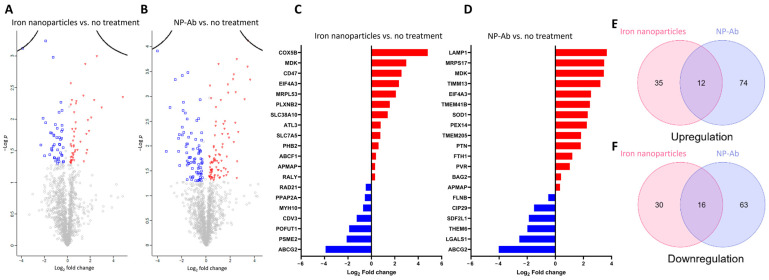
Comparisons of the proteome. (**A**,**B**) Volcano plot representing the log2 ratio for each protein quantified. The blue and red dots represent the downregulated and upregulated proteins, respectively. (**C**,**D**) The fold changes of the 20 most significantly differentially expressed proteins, present in treatment by both iron nanoparticles and NP-Ab. (**E**,**F**) Overlap of the upregulated and downregulated proteins in NP-Ab with iron nanoparticles compared to control cells.

In this study, label-free quantification (LFQ) was employed to measure the abundance of proteins. Following this, a total of 1398 proteins were quantified, with a false discovery rate of less than 1%, and were subsequently subjected to statistical sorting (Supplementary datasheet 1). The volcano plot depicted the differentially expressed proteins (DEPs) between the iron nanoparticles vs. no treatment and NP-Ab vs. no treatment (Figure 4A,B). The no treatment group served as a control, where cells were grown and extracted under the same conditions without any treatment. A list of all the DEPs can be found in Supplementary Tables S2 and S3. Figure 4C,D present the top 20 proteins with the greatest fold change for the two pairwise comparisons, iron nanoparticles vs. no treatment and NP-Ab vs. no treatment. Among the top 20 DEPs, 13 proteins were upregulated in the iron nanoparticle-treated cells compared to no treatment, including COX5B, MDK, CD47, EIF4A3, MRPL53, PLXNB2, SLC38A10, ATL3, SLC7A5, PHB2, ABCF1, APMAP, and RALY. Fourteen proteins were upregulated in the NP-Ab-treated cells compared to no treatment, such as LAMP1, MRPS17, MDK, TIMM13, EIF4A3, TMEM41B, SOD1, PEX14, TMEM205, PTN, FTH1, PVR, BAG2, and APMAP. Furthermore, when compared to the no treatment group, seven proteins in the iron nanoparticle-treated cells, including ABCG2, PSME2, POFUT1, CDV3, MYH10, PPAP2A, and RAD21, were downregulated. Among the top 20 DEPs, in the NP-Ab-treated cells, six were downregulated, including ABCG2, LGALS1, THEM6, SDF2L1, CIP29, and FLNB. Interestingly, this study found an overlap in the protein expression changes across the different nanoparticle treatments (Figure 4E,F). Among the overlapped proteins, 12 were upregulated, including APMAP, CD44, EIF4A3, FLOT2, IGFBP5, LUC7L2, MDK, PTN, RPL37, RPS23, TMEM205, and EMMPRIN, and 16 were downregulated, including ABCG2, CCT6A, CIP29, CSDA, CTTN, DCTN2, EEA1, HMGN2, KPNA2, LGALS1, NOC2L, OLA1, PPAP2A, RDX, SEPT2, and THEM6. A list of these overlapped proteins can be found in Supplementary Table S4.

#### Gene Ontology Analysis

**Figure 5:**
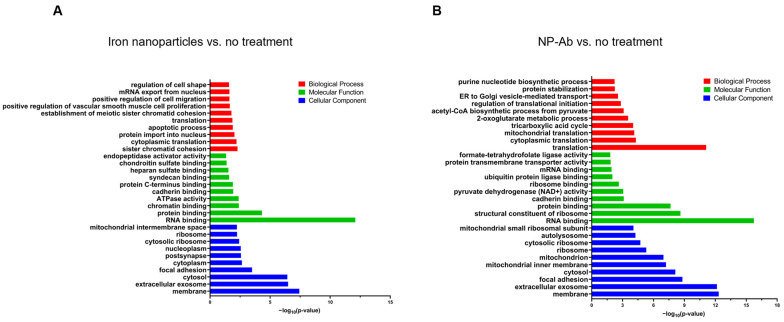
Gene ontology. Results from gene ontology analysis for the DEPs in iron nanoparticles vs. no treatment (**A**) and in NP-Ab vs. no treatment (**B**). The *p*-value (−log10-transformed) are represented on the graphs.

To determine the characteristics of the DEPs, we employed the GO enrichment analysis, which is categorized into three groups: biological process (BP), molecular function (MF), and cellular component (CC). The outcomes were sorted based on their enrichment score (EASE score), and we have presented the top 10 results for each category. In cases where there were limited outcomes to exhibit, we have included all significant enrichments (Figure 5 and Tables S5 and S6).

Between the iron nanoparticle and control groups, 93 DEPs showed most significant GO terms that were related to ‘sister chromatid cohesion’, ‘cytoplasmic translation’, ‘protein import into nucleus’, ‘apoptotic process’, ‘translation’, ‘establishment of meiotic sister chromatid cohesion’, ‘positive regulation of vascular smooth muscle cell proliferation’, ‘positive regulation of cell migration’, ‘mRNA export from nucleus’, and ‘regulation of cell shape’ in the BP category, ‘RNA binding’, ‘protein binding’, ‘chromatin binding’, ATPase activity’, ‘cadherin binding’, ‘protein C-terminus binding’, ‘syndecan binding’, ‘heparan sulfate binding’, ‘chondroitin sulfate binding’, and ‘endopeptidase activator activity’, in the MF category, and ‘membrane’, ‘extracellular exosome’, ‘cytosol’, ‘focal adhesion’, ‘cytoplasm’, ‘postsynapse’, ‘nucleoplasm’, ‘cytosolic ribosome’, ‘ribosome’, and ‘mitochondrial intermembrane space’ in the CC category (Figure 5A).

GO analysis of the DEPs between the NP-Ab and control groups yielded the top 10 most significant results for ‘translation’, ‘cytoplasmic translation’, ‘mitochondrial translation’, ‘tricarboxylic acid cycle’, ‘2-oxoglutarate metabolic process’, ‘acetyl-CoA biosynthetic process from pyruvate’, ‘regulation of translational initiation’, ‘ER to Golgi vesicle-mediated transport’, ‘protein stabilization’, and ‘purine nucleotide biosynthetic process’ in the BP, ‘RNA binding’, ‘structural constituent of ribosome’, ‘protein binding’, ‘cadherin binding’, ‘pyruvate dehydrogenase (NAD+) activity’, ‘ribosome binding’, ‘ubiquitin protein ligase binding’, ‘mRNA binding’, ‘protein transmembrane transporter activity’, and ‘formate-tetrahydrofolate ligase activity’ in the MF, and ‘membrane’, ‘extracellular exosome’, ‘focal adhesion’, ‘cytosol’, ‘mitochondrial inner membrane’, ‘mitochondrion’, ‘ribosome’, ‘cytosolic ribosome’, ‘autolysosome’, and ‘mitochondrial small ribosomal subunit’ in the CC categories, as presented in Figure 5B.

We employed ingenuity pathway analysis (IPA) to analyse the canonical pathways, which were evaluated based on the Fisher’s exact test (*p*-value < 0.05). The affected canonical pathways are shown in Figure S4, along with the *p*-value and z-score, which is an algorithm that is used to predict more active functions. Additionally, we conducted an analysis of the overlapping canonical pathways between the iron nanoparticles vs. control groups and NP-Ab vs. control groups, which were categorized as ‘Mitochondrial Dysfunction’, as shown in Figure S6.

To better understand the interaction between these DEPs, we employed a protein–protein interaction (PPI) network analysis using the STRING database (Figure S7). The results from STRING indicated that these DEPs were more significantly interacted than expected for a random set of a similar size drawn from the genome (enrichment *p*-value < 0.05).

## 4. Discussion

A timely diagnosis of AD is necessary to ensure that patients benefit the most from therapeutic interventions earlier in the disease progression. Our project aimed to conduct a preliminary investigation of a nanoformulation developed to enhance MRI contrast between the amyloid plaques and the parenchyma in AD. This would enable an earlier imaging diagnosis of AD as an alternative to PET, which is less widely accessible than MRI and requires the use of a radioactive tracer. The recent FDA approval of the AD drugs aducanumab and lecanemab has amplified the urgent need for an accessible method for confirming the presence of *Aβ* plaques in the brain. This would permit patients access to these disease-modifying treatments or, on the other hand, prevent misdiagnosis and provide more certainty around the prescription of these and future medications. The iron nanoparticles in our study are tailored for neurodiagnostic assessments, as they are able to achieve high saturation magnetization while keeping the core size small, a property essential for promoting blood–brain barrier permeation [51]. The addition of a DMSA coating permits their functionalization with an *Aβ*-targeting ligand, while maintaining their stability and biocompatibility.

As a major direct indicator for expected MRI performance, we measured the *r*_2_ relaxivity of our samples. Briefly, the expected signal change depends on the product of signal change per particle (as determined by its relaxivity) and the local particle density. The measured *r*_2_ relaxitivity of our particle without functionalization was 129.29 mM^−1^s^−1^, which is slightly lower than the *r*_2_ of Resovist (~180 mM^−1^s^−1^), which was used as a commercial reference. One possible reason for this is the currently thicker coating of our particles. This is something which will be addressed in future research; however, the focus of this study was to synthesize particles which can be directly functionalized—a feature not available on the commercial counterpart. Expectedly, the addition of antibodies to the surface decreased the *r*_2_ values in magnetic nanoparticles by increasing the distance between the water molecules and the metal centre, among other mechanisms. In our nanoparticles, relaxivity reduces from *r*_2_ = 129.29 mM^−1^s^−1^ to *r*_2_ = 55.99 mM^−1^s^−1^ when the antibodies were conjugated. Binding studies demonstrated the NP-Ab binding to the A*β*_(1–42)_ peptide; although, their binding affinities were lower than that of the antibodies alone. Further developments will be necessary to improve the sensitivity of the contrast agents to require lower doses while maintaining an effective MRI signal.

To ensure the clinical success of future nanoformulations, it is essential to comprehensively evaluate and describe the in vitro and in vivo behaviour of nanoparticles and possible mechanisms of toxicity and elimination routes. In our study, following 24 h of treatment, cell viability was initially evaluated using the lactate dehydrogenase assay. Cell viability remained high, even at the highest concentrations of iron nanoparticles and NP-Ab. Interestingly, functionalization with antibodies resulted in a decreased relative lactate dehydrogenase level at the highest concentration of 500 µM. However, the protective effect of anti-A*β* antibodies on cell survival has been primarily explored as a response to toxicity induced by A*β* [52,53]. IgG conjugated to gold nanoparticles, as well as intravenous immunoglobulin therapy at high concentrations were both previously shown to exert anti-inflammatory effects on cells [54]. Intravenous immunoglobulin therapy, which contains naturally occurring autoantibodies against A*β*, protects cortical neurons against A*β*-induced cell death by downregulating the c-Jun NH_2_ terminal kinase (JNK) pathway, which is activated during times of cellular stress, and the NF-κB signalling pathway [54]. Although the scope of this study was to develop a targeted diagnostic agent, this result points to the additional possibility of the therapeutic use of these nanomaterials. One of the primary challenges of AD therapeutics has been the delivery of an effective dose across the blood–brain barrier. The surface area of nanoparticles allows multiple antibodies to decorate the surface, which can facilitate a higher therapeutic dose reaching targets. Further work should explore the possible protective effect observed at high concentrations of the NP-Ab. For subsequent analyses in the present study, the lowest effective MRI contrast-enhancing dose of 100 µM of iron nanoparticles and NP-Ab was used.

Electron microscopy results demonstrated that both the iron nanoparticles and conjugated NP-Ab were readily endocytosed by glioblastoma cells within 5 min of incubation (Figure 2). Membrane protrusions (Figure 2A, double arrows) form during macropinocytosis, a type of endocytosis which has been shown as the internalization mechanism for various nanoparticles [55,56,57], particularly for agglomerates of particles around 200 nm in size, corresponding to our observations. Generally, it has been demonstrated that the clustering of nanoparticles is more favourable for cell uptake at diameters below 30–40 nm, as the wrapping of individual nanoparticles is not possible due to the associated high bending energy [58]. Following internalization, all nanoparticles were localized within membrane-bound organelles, namely the endosomes and lysosomes, following a route through the cell which has been observed in similar nanoparticles [59], those with different coatings (e.g., PEG, PEI, and carboxylate), and across cell types (macrophages and cancer cells) [60,61,62], as well as in vivo within phagocytes in the spleen, liver, and adipose tissue [63].

Proteomics is a high-throughput technology which allows further insight into nanoparticle interactions with the cellular environment. The aspects of the proteome which are affected depend on their material composition, size, route of administration, and the cells or tissues used for the analysis. Previous studies looking at the proteomic changes induced by magnetic nanoparticles have looked at NRK-52E cell lines [64], SH-SY5Y cells [65], Jurkat cells [66], and HeLa cells [67]. However, other studies have used nanoparticles as cancer-targeting agents, wherein cellular toxicity was the aim, and the nanoparticles were used in higher concentrations and lacked a biocompatible coating. This is the first study looking at the effects of antibody-functionalized and non-functionalized iron nanoparticles on U-251 glioblastoma cells. A relatively modest proteomic change was observed in response to treatment with the nanoparticles, and among the most significantly deregulated proteins were those related to energy metabolism and protein synthesis and turnover. Interaction with an external stimulus is energy-demanding, and mitochondrial translation and mitochondrial ribosomal proteins were enriched in cells treated with NP-Ab and iron nanoparticles. In addition, proteins involved in stress signalling pathways were altered in response to both treatments.

The existence of endogenous iron processing and recycling mechanisms within cells lends itself to the biocompatibility of iron nanoparticles. In our study, multiple DEPs following both treatments (iron nanoparticles: HNRNPH2, HIST2H3A, RPS23, HMGN2, LGALS1; NP-Ab: RPS23, HMGN2, LGALS1, and FEN1) corresponded to proteins that have been previously shown to be altered in neuroblastoma cells treated with FeCl_3_ [65], suggesting alterations to ferric iron levels in the cells and some nanoparticle degradation occurring at 24 h post-treatment. NP-Ab-treated cells showed upregulated ferritin H (FTH1) and ferritin L (FTL), which make up the intracellular ferritin complex, and are key proteins in iron metabolism and homeostasis. Higher incubation times and doses impact FTH/FTL expression levels, with DMSA-coated iron oxide nanoparticles previously being shown to upregulate these proteins [68,69], while others induced no changes [39]. Downregulation of FTH can result in ferroptosis, as this protein converts Fe^+2^ into Fe^+3^ and prevents the production of ROS. Magnetic nanoparticles localizing in the lysosomes as part of the degradation pathway decompose to free iron ions [70,71], which may accumulate and disrupt mitochondrial functioning or generate singlet oxygen through the Fenton reaction leading to cellular injury, resulting in heightened ROS production. The non-functionalized iron nanoparticles in this study did not alter FTH/FTL expression, which may be due to the lower iron load internalized after 24 h compared to the NP-Ab. In addition, iron nanoparticles did not induce ROS production in U-251 cells, nor did they generate superoxide at any of the concentrations evaluated, as determined using the ROS/superoxide detection kit (Figure S5). Oxidative stress results from the generation of superoxide and other ROS, such as hyperoxide, singlet oxygen, and peroxide from free neutral oxygen, in a reaction catalysed by oxidoreductases [72]. Correspondingly, proteins involved in oxidoreductase activity were downregulated in the iron nanoparticle-treated cells (NDUFB11 and IDH3B), while proteins in the NP-Ab-treated cells demonstrated both downregulation (MTHFD1 and IDH3B) and upregulation (COX5B, NNT, and TMX1).

Protein changes related to cytoskeletal organisation and cell migration and motility correspond to the potential internalization mechanisms observed with TEM. Downregulation of the src substrate cortactin (CTTN), pleiotrophin (PTN), and upregulation of the hyaluronic acid receptor (CD44) and RDX was common to treatments with both iron nanoparticles and NP-Ab. Cytoskeletal organization and cell shape are regulated by PTN, which exerts its effects through the ligation of the cell surface-binding protein N syndecan, resulting in the phosphorylation of src and CTTN [73]. In addition, CTTN is a downstream transcriptional target of CD44 [74], a protein involved in clathrin-independent endocytosis. Taken together, these results suggest cytoskeletal reorganization in response to nanoparticle treatment. Previous research has shown that cell motility, which is important for the regeneration and maintenance of tissues, can be affected by iron nanoparticle treatment, potentially due to the tensile force of nanoparticle internalization into cells [75]. However, its impact on cell motility is dose-dependent [76] and transient, with cells recovering following the elimination of iron through either cell division [77] or exocytosis [78]. Indeed, the perturbations to the cytoskeletal organization proteins suggest the migration of the particles into cells during the incubation period in this study, rather than deleterious effects on the cells. Unlike other forms of endocytosis, macropinocytosis, which can be observed in the TEM micrographs at all timepoints, requires actin cytoskeleton reorganisation [79] to develop actin-rich membrane protrusions. In addition, proteomic studies using other nanoparticles, such as Resovist and Endorem [80], also reported cytoskeletal disturbance. Nevertheless, these data necessitate a thorough further investigation of the optimal dose which does not exert adverse effects on cell motility.

Following endocytosis, nanoparticles may be trafficked towards lysosomes via endosomes, either for degradation or lysosomal exocytosis [81]. Following 24 h of incubation with both NP-Ab and iron nanoparticles, EEA1, which is essential for early endosome binding and fusion [82], was downregulated. Interestingly, CD44, which was upregulated, is implicated in cell trafficking mechanisms that avoid the degradative pathway downstream of the EEA1-positive endosomes [83]. On the other hand, adipocyte plasma membrane-associated protein (APMAP), a gene involved in the autophagy–lysosome system [84], was upregulated following both treatments, and LAMP1 was only upregulated in the NP-Ab-treated cells. Although this was in contrast to the lower LAMP1 expression observed with immunofluorescence (Figure 2D,E), the merged LAMP1 fluorescence and brightfield images (Figure 2C) showed nanoparticle agglomerates obscuring the fluorescent signal, which potentially impacted the quantification of the signal. Fourteen other deregulated lysosomal proteins were discovered through proteomic analysis in the NP-Ab-treated cells and 49 exosomal proteins, further implicating the lysosomal processing of the NP-Ab (Table S6). Common to both treatments was the downregulation of ATP-binding cassette sub-family G member 2 (ABCG2), a translocation protein expressed in cells in the blood–brain barrier (BBB), which regulates the drug efflux properties of the BBB [85,86]. Interestingly, ABCG2 is also implicated in iron uptake and regulation through its involvement in heme secretion from cells [87]. Further analysis of exosomal cargo would permit further elucidation of the processing and exocytosis of the conjugated nanoparticles to ensure that they can reach their target in the brain prior to degradation. Other methods of studying nanoparticle passage through cells include the use of chemical inhibitors or the silencing of genes involved in cell uptake and transport; however, these induce responses in cells that are not true to physiological conditions, highlighting the crucial role of quantitative proteomics in the study of the nanoparticle interaction with cells.

The endoplasmic reticulum of cells is very sensitive to extracellular stimuli, and certain events, such as nutrient deprivation, oxidative stress, or saturation of protein folding, can result in endoplasmic reticulum stress. Exposure to nanoparticles, such as iron oxide nanoparticles, gadolinium-based contrast agents, and manganese oxide nanoparticles, has been shown to induce endoplasmic reticulum stress in mice [88]. Similarly, pro-apoptotic genes and inflammatory pathways were activated in cells following exposure to silver nanoparticles [89]. In the present study, the unfolded protein response protein Wolframin (WFS1), as well as other proteins involved in endoplasmic reticulum stress response (GORAPS2, FLOT1, and HYOU1) were upregulated in the NP-Ab-treated cells. In fact, WFS1 inhibits cell death pathways via negative regulation of endoplasmic reticulum stress in cells. Other antiapoptotic proteins, including NNT, VDAC1, SQSTM1, and MDK, were also upregulated in the NP-Ab-treated cells. Given that no decrease in cell viability was observed in the lactate dehydrogenase assay, these results suggest that the cells used in this study underwent an expected degree of stress in response to the internalization of exogenous agents, but successfully initiated compensatory pathways. Iron nanoparticle-treated cells showed enrichment in apoptotic process regulation, including the upregulation of AIFM1, which is released from the mitochondria during apoptotic processes [90]; conversely, BCL2-associated transcription factor 1 (BCLAF-1), whose overexpression has been shown to trigger apoptosis [91], was downregulated.

This study provided an initial in vitro bioevaluation of novel iron nanoparticles and targeted nanoparticles for the diagnosis of AD. The cellular data support the biocompatibility of these conjugates; however, the cell lines used were not representative of in vivo conditions; moreover, only one cell type was evaluated in this study. It is essential to complete in vivo assessments of nanoparticle safety and efficacy, as well as their blood circulation time and stability. Proteomic investigations demonstrated the pathways implicated in cellular internalization and response to nanoparticle treatment. While the predominant proteomic changes involved energy metabolism, protein turnover, and cytoskeletal reorganization, these results also present the potential pathways of cytotoxicity and cellular stress. To ensure the successful clinical translation of the described nanoparticles, these pathways must be investigated further, and the dosing optimized to improve biocompatibility. Furthermore, a major challenge for neurodiagnostic and neurotherapeutic materials is achieving a sufficient crossing of the blood–brain barrier. As the blood–brain barrier is composed of endothelial cells, pericytes, and astrocytes [92], the endocytosis of nanoparticles using a glial cell line, and in particular the increased internalization of the surface-modified NP-Ab, suggests that the nanoparticles in this study could be capable of crossing the blood–brain barrier into the brain parenchyma. It has been shown that albumin-modified DMSA-coated nanoparticles are able to cross the blood–brain barrier [93]. Future work is required to determine the capacity and mechanism of the nanoparticles to pass through the blood–brain barrier into the brain.

## 5. Conclusions

In this work, we propose an approach to improve the sensitivity of current neuroimaging methods for the early diagnosis of AD. We present novel iron core-iron oxide core shell nanoparticles targeted to A*β* for their use as T2-weighted MRI contrast agents. The DMSA-coated iron nanoparticles and A*β*-targeted NP-Ab demonstrated in vitro biocompatibility, as determined using cellular assays and discovery proteomics, as well as favourable MRI relaxivity (129.29 mM^−1^s^−1^ and 55.99 mM^−1^s^−1^, respectively). TEM and ICP-MS studies demonstrated that the nanoparticles were readily internalized by glioblastoma cells and localized within cellular organelles. Proteomics results indicated that cells initiate compensatory mechanisms to respond to nanoparticle internalization, with enrichment observed in proteins implicated in energy metabolism and translation processes, and in the case of the NP-Ab, in the iron storage machinery. The NP-Ab were able to effectively stain ex vivo A*β* plaques and demonstrated their binding affinity for the A*β*_(1–42)_ peptide. However, the addition of the targeting ligand increased the hydrodynamic size (369.5 nm) and reduced the MRI contrast-enhancing properties of the nanoparticles. Future work should focus on improving the sensitivity of the particles, while maintaining their stability in biological media, as well as assessing their efficacy and biocompatibility in an animal model. Early diagnosis is imperative for the successful treatment of neurodegenerative diseases, and contrast-enhanced MRI is a promising and powerful early detection tool. The results presented here demonstrate the excellent potential of biocompatible iron nanoparticles targeted at amyloid pathology for use in AD diagnosis.

## Figures and Tables

**Table 1:** Characterization of nanoparticles. Size, polydispersity index (PDI), and zeta potential were measured in MilliQ water at 25 °C.

	TEM Diameter (nm)	Hydrodynamic Size (nm)	PDI	Zeta Potential (mV)
Iron nanoparticles	11.6 ± 0.9	106.7	0.141	−22.7 ± 6.26
NP-Ab		369.5	0.994	−31.5 ± 4.12

## Data Availability

The data presented in this study are available in the present article and Supplementary Materials: “Supplementary materials”, “Supplementary Datasheet 1”.

## Supplementary Materials

Supporting information can be downloaded at: https://www.mdpi.com/article/10.3390/cells12182279/s1. Figure S1: (A) Transmission electron micrograph (TEM) of iron nanoparticles. (B) Size distribution of dry diameter. (C) High resolution TEM image showing the darker crystalline core, and lighter oxide shell of one iron nanoparticle. (D,E) DLS and zeta potential of iron nanoparticle (D) and NP-Ab (E); Figure S2: Micrographs from a light microscope of adjacent brain sections of APP/PS1 mice immunostained for amyloid-beta plaques with NP-Ab (A,B) and an anti-amyloid-beta antibody (C,D) as control. DAB was used as a chromogen and plaques appear as dark brown circles on image. Plaques present in corresponding regions on control section confirmed presence of antibodies on nanoparticles. Figure S3: Binding affinity of anti-A*β* antibody to A*β*(1–42) peptide immobilised CM5 sensor different concentrations. Binding kinetics of anti- A*β* antibody to A*β*(1–42) peptide were monitored by BiaCore T200. The data were fitted to a 1:1 binding model to calculate the equilibrium dissociation constant (KD); Figure S4: Binding affinity of NP-Ab to ProG sensors at different concentrations. (A) NP-Ab binding to ProG sensors. (B) Kinetics of A*β*(1–42) peptide binding to NP-Ab captured ProG sensors (colored lines). The data were fitted to a 1:1 model to calculate the equilibrium dissociation constant (KD); Figure S5: Level of reactive oxygen species (ROS) and superoxide production in U-251 cells following 24 h treatment with iron nanoparticles at three different concentrations. Pyocyanin was used as a positive control ROS inducer, Milli Q water was used as a vehicle control. Data represent mean ± SD (*n* = 5), *: significant difference from positive control (*p* < 0.05); Figure S6: Canonical pathways of iron nanoparticle vs. no treatment (A), NP-Ab vs. no treatment (B), and comparison between groups (C); Figure S7: STRING diagrams of iron nanoparticle vs. no treatment (A,B), NP-Ab vs. no treatment (C,D), down (A,C) and up (B,D) regulated proteins; Table S1: Mutagenicity of iron nanoparticles and NP-Ab in Salmonella typhirium. *: *p* < 0.05 based on reference table https://www.biotoxicity.com/images/Toxicity%20PDF/muta-ChromoPlate_Ames_Test.pdf (accessed on 9 August 2023); Table S2: List of significantly differentially expressed proteins in Iron nanoparticle vs. no treatment; Table S3: List of significantly differentially expressed proteins in NP-Ab vs. no treatment; Table S4: List of overlapped DEPs across different nanoparticle treatment (iron nanoparticles and NP-Ab); Table S5: Gene ontology enrichment analysis of DEPs (Iron nanoparticle vs. control); Table S6: Gene ontology enrichment analysis of DEPs (NP-Ab vs. control).
