# Supplementary material for: Evaluation of Dimercaptosuccinic Acid-Coated Iron Nanoparticles Immunotargeted to Amyloid Beta as MRI Contrast Agents for the Diagnosis of Alzheimer’s Disease
Source: Cells. 2023 Sep 14;12(18):2279. doi: 10.3390/cells12182279 (PMC10527350; doi:10.3390/cells12182279)
Supplement: Supplementary file 1 [file cells-12-02279-s001.zip › Supplementary materials.pdf]

Electronic supplementary information

Evaluation of DMSA-coated iron nanoparticles immunotargeted to amyloid-beta as MRI contrast agents for the diagnosis of Alzheimer's disease

Marina Ulanova, Lucy Gloag, Andre Bongers, Chul-Kyu Kim, Hong Thien Kim Duong, Ha Na Kim, J. Justin Gooding, Richard D. Tilley, Joanna Biazik, Wei Wen, Perminder S. Sachdev, Nady Braidy*

Supplementary method

Surface Plasmon Resonance Analysis for Anti-Amyloid Antibody and Aβ(1-42) Peptide Binding

BiaCore T200 (Cytiva) was used to measure the binding kinetic of $A\beta_{(1\text{-}42)}$ peptide (Sigma A9810) to anti-amyloid antibody. $A\beta_{(1\text{-}42)}$ peptide was immobilised on a CM5 sensor using amine coupling chemistry. Prior to the ligand immobilisation step, BiaCore T200 was primed with the immobilisation running buffer, HBS-EP (0.15 M NaCl, 10 mM HEPES, 3 mM EDTA, 0.005 % (v/v) P20 surfactant, pH 7.4). The surfaces of two flow cells were activated for 7 min with a 1:1 mixture of 0.1 M NHS (N-hydroxysuccinimide) and 0.1 M EDC (3-(N, N-dimethylamino) propyl-N-ethylcarbodiimide) at a flow rate of 10 µL/min. The $A\beta_{(1\text{-}42)}$ peptide at a concentration of 4 µM in 10 mM sodium acetate, pH 4 was injected on flow cell 2 of the activated sensor surface for 7 min. 95.4 RU of ligand density was achieved on flow cell 2, whereas flow cell 1 was left blank to be used as a reference channel. The unbound sensor surfaces were blocked with 7 min injection of 1 M ethanolamine, pH 8.

For kinetic measurement, anti-amyloid antibody in HBS-EP+ (0.15 M NaCl, 10 mM HEPES, 3 mM EDTA, 0.05 % (v/v) P20 surfactant, pH 7.4) was injected at concentrations of 1.2, 3.6, 11.11, 33.33, 100 nM to the ligand immobilised sensor at a flowrate of 30 µL/min. The association and dissociation of anti-amyloid antibody to $A\beta_{(1\text{-}42)}$ peptide were measured for 120 s and 480 s, respectively. The sensor surface was regenerated using 10 mM glycine-HCl, pH 2. The collected data were fit to 1:1 interaction model using the global data analysis in Biacore T200 Evaluation Software.

Biolayer Interferometry Analysis for NP-Ab and Aβ(1-42) Peptide Binding

Kinetic binding of $A\beta_{(1\text{-}42)}$ peptide (Sigma A9810) to NP-Ab was assessed using a BLItz system, which is a label-free biosensor technology that measures protein-protein interactions in real-time. BLItz Protein G (ProG) biosensors were incubated in running buffer (PBS containing 0.005 % Tween-20) for a minimum of 10 min to dissolve the sucrose coating on the biosensors. The baseline of the activated BLItz ProG biosensor was established with the running buffer for 30 seconds followed by dipping into the NP-Ab sample to capture nanoparticles for 180 seconds. $A\beta_{(1\text{-}42)}$ was diluted in the running buffer at 1, 4 and 10 µM, and the NP-Ab captured ProG biosensor tip was then dipped into $A\beta_{(1\text{-}42)}$ for 180 seconds, followed by a dissociation step in the running buffer for 120 seconds. The association and dissociation of each interaction were recorded in real-time. The association and dissociation rate constants (ka and kd), equilibrium dissociation constant (KD), and maximum binding response (Δnm) were calculated for each interaction.

	**Step Type**	**Duration (s)**	**Position**
**1**	Initial Baseline	30	Tube
**2**	Loading (NP-Ab)	180	Drop
**3**	Baseline	30	Tube
**4**	Association (Peptides)	180	Drop
**5**	Dissociation	120	Tube

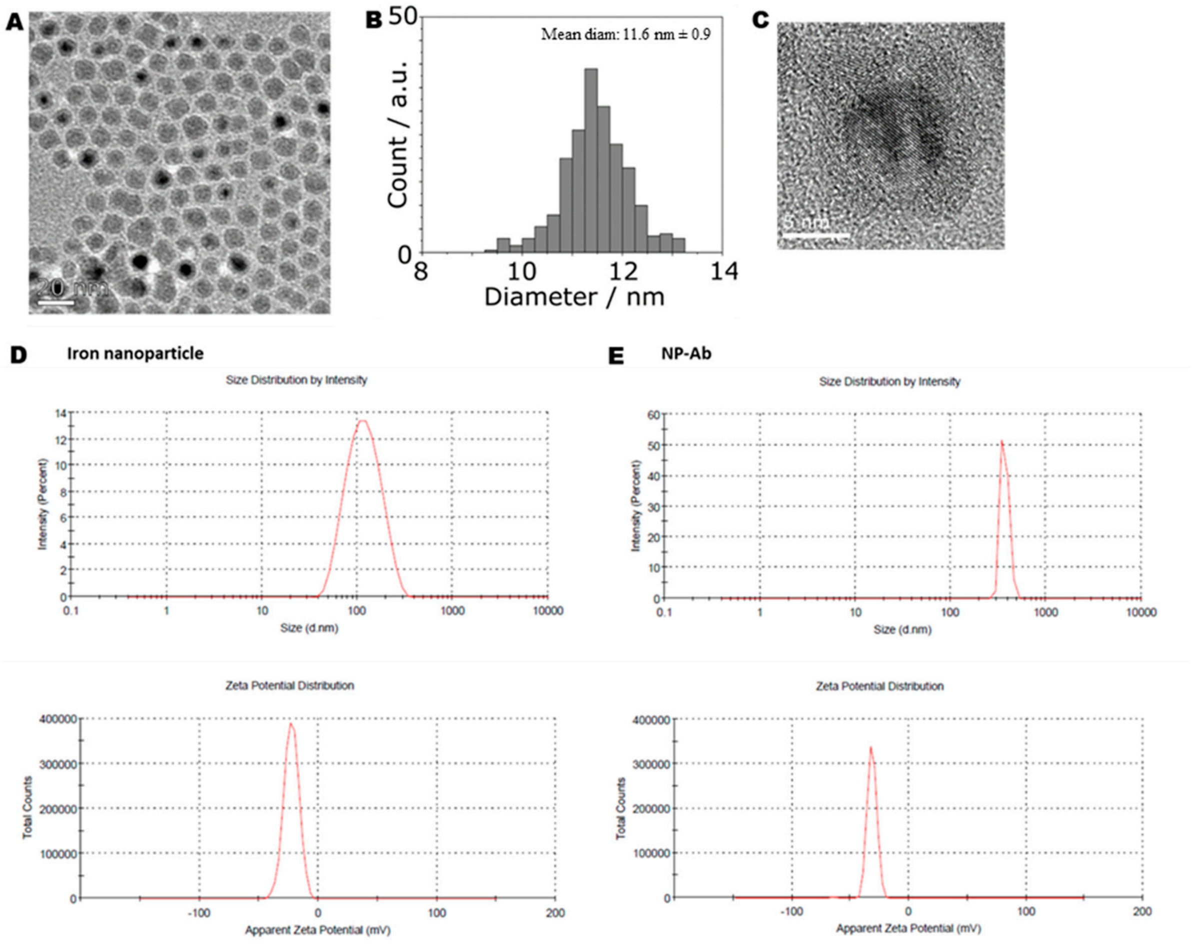

Figure S1. (A) Transmission electron micrograph (TEM) of iron nanoparticles. **(B)** Size distribution of dry diameter. **(C)** High resolution TEM image showing the darker crystalline core, and lighter oxide shell of one iron nanoparticles. **(D, E)** DLS and zeta potential of iron nanoparticles **(D)** and NP-Ab **(E)**

.

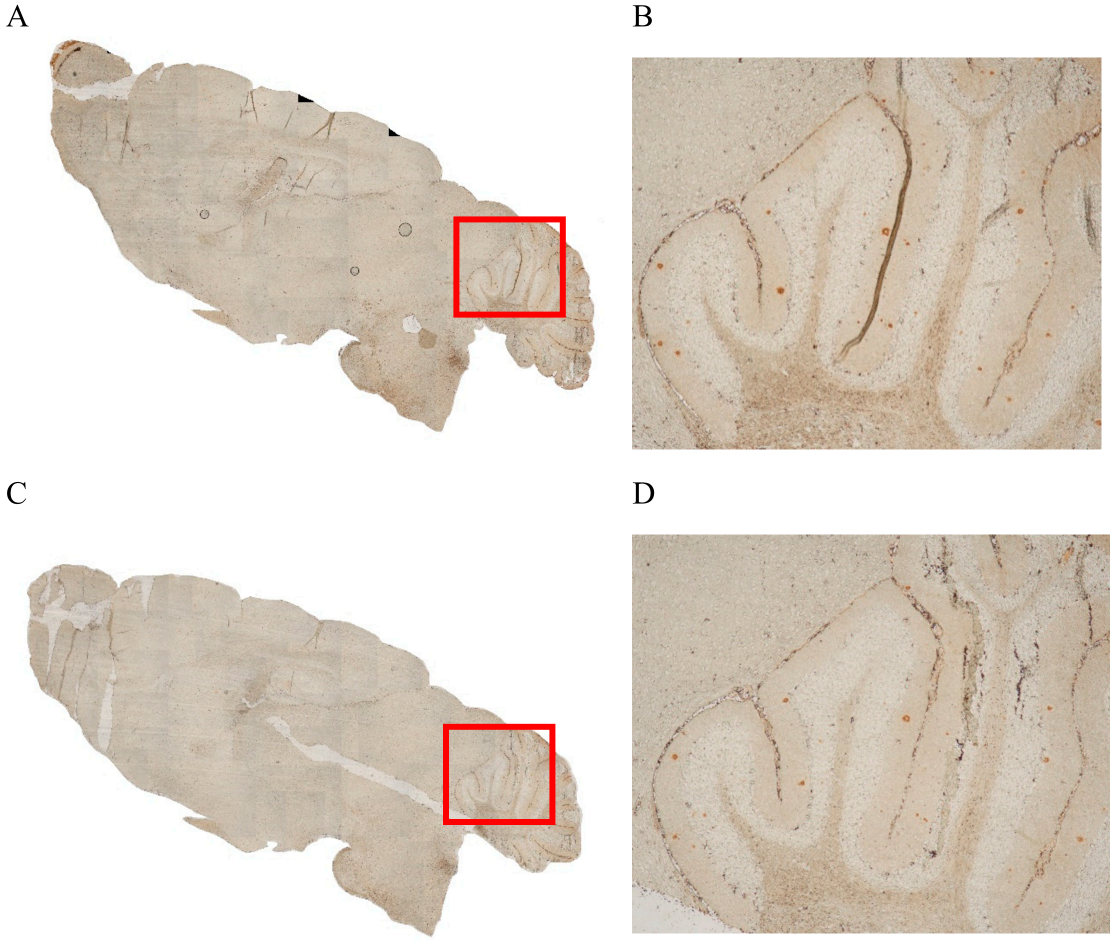

Figure S2. Micrographs from a light microscope of adjacent brain sections of APP/PS1 mice immunostained for amyloid-beta plaques with NP-Ab (A, B) and an anti-amyloid-beta antibody (C, D) as control. DAB was used as a chromogen and plaques appear as dark brown circles on image. Plaques present in corresponding regions on control section confirmed presence of antibodies on nanoparticles.

Conc (nM)	KD (M)	ka (1/Ms)	kd (1/s)
Global fitting			
1.2	2.14E-09	1.44E+05	3.08E-04
3.6			
11.11			
33.33			
100			

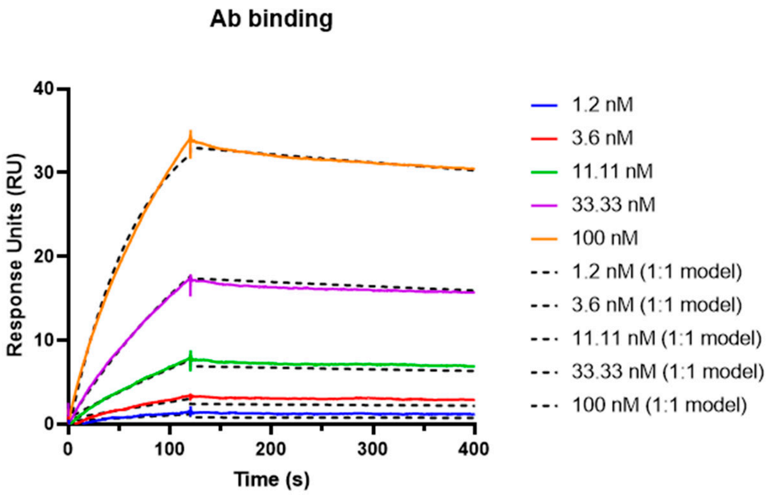

Figure S3. Binding affinity of anti-Aβ antibody to A$\beta_{(1-42)}$ peptide immobilised CM5 sensor different concentrations. Binding kinetics of anti- Aβ antibody to A$\beta_{(1-42)}$ peptide were monitored by BiaCore T200. The data were fitted to a 1:1 binding model to calculate the equilibrium dissociation constant (KD).

A

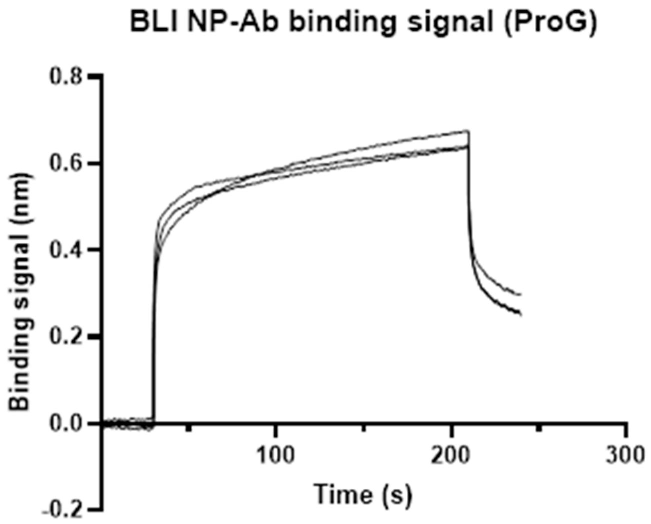

B

Conc (uM)	KD (M)	ka (1/Ms)	kd (1/s)
Global fitting			
1	2.395E-6	4.288E3	1.027E-2
4			
10			

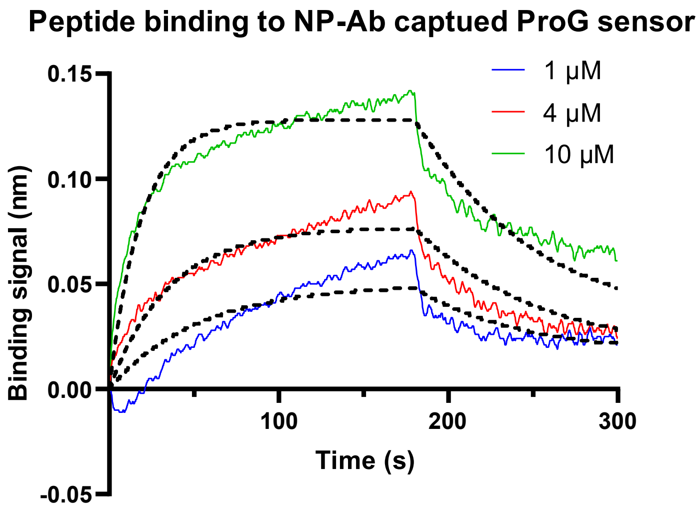

Figure S4. Binding affinity of NP-Ab to ProG sensors at different concentrations. **(A)** NP-Ab binding to ProG sensors. **(B)** Kinetics of A$\beta_{(1\text{-}42)}$ peptide binding to NP-Ab captured ProG sensors (colored lines). The data were fitted to a 1:1 model to calculate the equilibrium dissociation constant (KD).

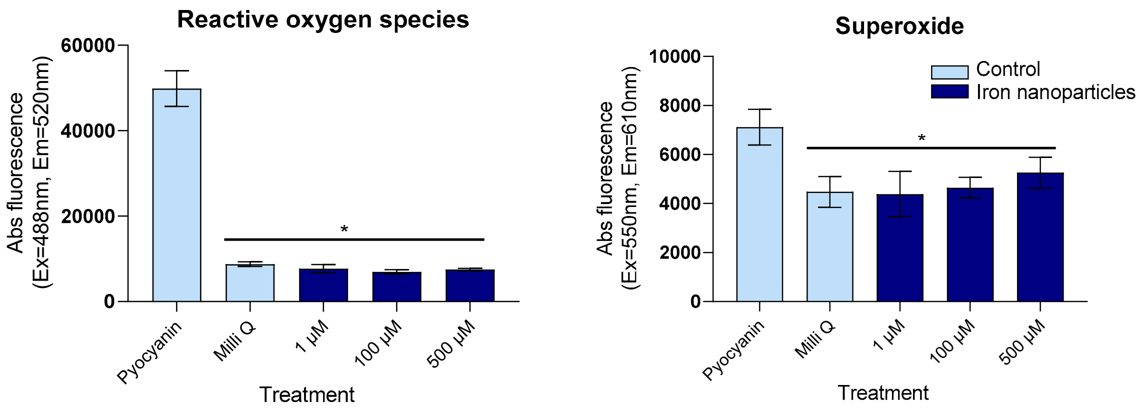

Figure S5. Level of reactive oxygen species (ROS) and superoxide production in U-251 cells following 24 h treatment with iron nanoparticles at three different concentrations. Pyocyanin was used as a positive control ROS inducer, Milli Q water was used as a vehicle control. Data represent mean ± SD (n = 5), *: significant difference from positive control ($p < 0.05$).

Table S1 Mutagenicity of iron nanoparticles and NP-Ab in *Salmonella typhirium*. *: $p < 0.05$ based on reference table https://www.biotoxicity.com/images/Toxicity%20PDF/muta-ChromoPlate_Ames_Test.pdf.

	Number of positive wells		Total testable wells (48 - excluded wells)		Proportion of positive wells (%)			
Treatment	Sample 1	Sample 2	Sample 1	Sample 2	Sample 1	Sample 2	Average (%)	SD
Negative control	2	3	48	48	4.17	6.25	5.21	1.47
Positive control	48	40	48	40	100.00	100.00	100.00	0.00
50 µM Iron nanoparticle	5	6	45	40	5.33	15.00	10.17	6.84
100 µM Iron nanoparticle	11	10	37	37	14.27	27.03	**20.65***	9.02
50 µM NP-Ab	7	4	33	29	10.18	13.79	11.99	2.55

A

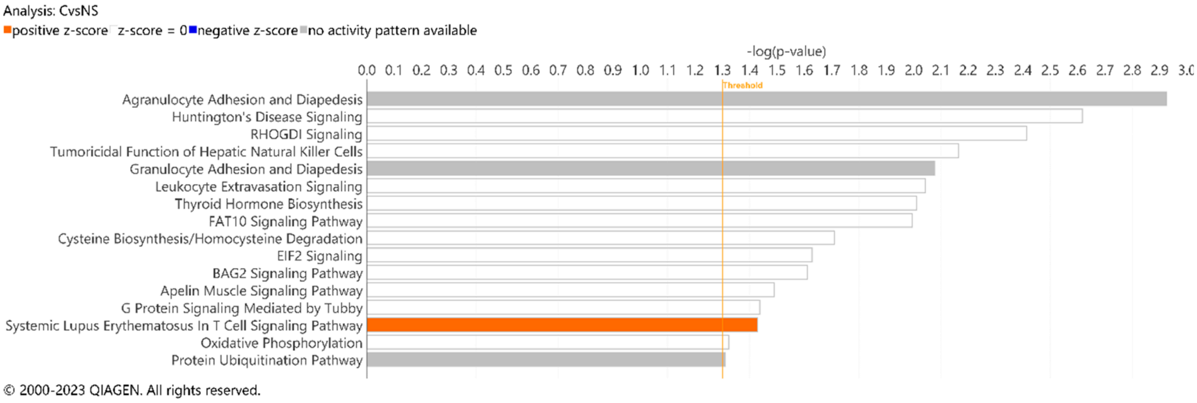
Analysis: CvsNS
positive z-score
z-score = 0
negative z-score
no activity pattern available
-log(p-value)
0.0 0.1 0.2 0.3 0.4 0.5 0.6 0.7 0.8 0.9 1.0 1.1 1.2 1.3 1.4 1.5 1.6 1.7 1.8 1.9 2.0 2.1 2.2 2.3 2.4 2.5 2.6 2.7 2.8 2.9 3.0
Threshold
Agranulocyte Adhesion and Diapedesis
Huntington's Disease Signaling
RHOGDI Signaling
Tumoricidal Function of Hepatic Natural Killer Cells
Granulocyte Adhesion and Diapedesis
Leukocyte Extravasation Signaling
Thyroid Hormone Biosynthesis
FAT10 Signaling Pathway
Cysteine Biosynthesis/Homocysteine Degradation
EIF2 Signaling
BAG2 Signaling Pathway
Apelin Muscle Signaling Pathway
G Protein Signaling Mediated by Tubby
Systemic Lupus Erythematosus In T Cell Signaling Pathway
Oxidative Phosphorylation
Protein Ubiquitination Pathway
© 2000-2023 QIAGEN. All rights reserved.

B

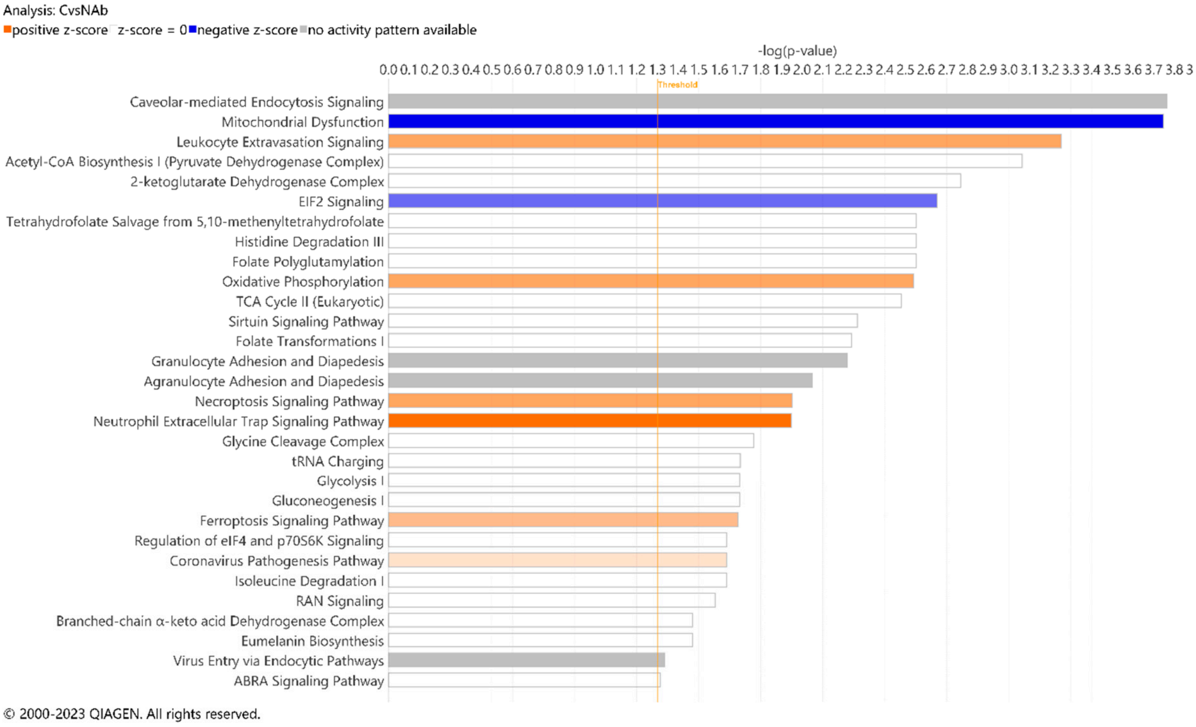
Analysis: CvsNAb
positive z-score
z-score = 0
negative z-score
no activity pattern available
-log(p-value)
0.0 0.1 0.2 0.3 0.4 0.5 0.6 0.7 0.8 0.9 1.0 1.1 1.2 1.3 1.4 1.5 1.6 1.7 1.8 1.9 2.0 2.1 2.2 2.3 2.4 2.5 2.6 2.7 2.8 2.9 3.0 3.1 3.2 3.3 3.4 3.5 3.6 3.7 3.8
Threshold
Caveolar-mediated Endocytosis Signaling
Mitochondrial Dysfunction
Leukocyte Extravasation Signaling
Acetyl-CoA Biosynthesis I (Pyruvate Dehydrogenase Complex)
2-ketoglutarate Dehydrogenase Complex
EIF2 Signaling
Tetrahydrofolate Salvage from 5,10-methenyltetrahydrofolate
Histidine Degradation III
Folate Polyglutamylation
Oxidative Phosphorylation
TCA Cycle II (Eukaryotic)
Sirtuin Signaling Pathway
Folate Transformations I
Granulocyte Adhesion and Diapedesis
Agranulocyte Adhesion and Diapedesis
Necroptosis Signaling Pathway
Neutrophil Extracellular Trap Signaling Pathway
Glycine Cleavage Complex
tRNA Charging
Glycolysis I
Gluconeogenesis I
Ferroptosis Signaling Pathway
Regulation of eIF4 and p70S6K Signaling
Coronavirus Pathogenesis Pathway
Isoleucine Degradation I
RAN Signaling
Branched-chain α-keto acid Dehydrogenase Complex
Eumelanin Biosynthesis
Virus Entry via Endocytic Pathways
ABRA Signaling Pathway
© 2000-2023 QIAGEN. All rights reserved.

C

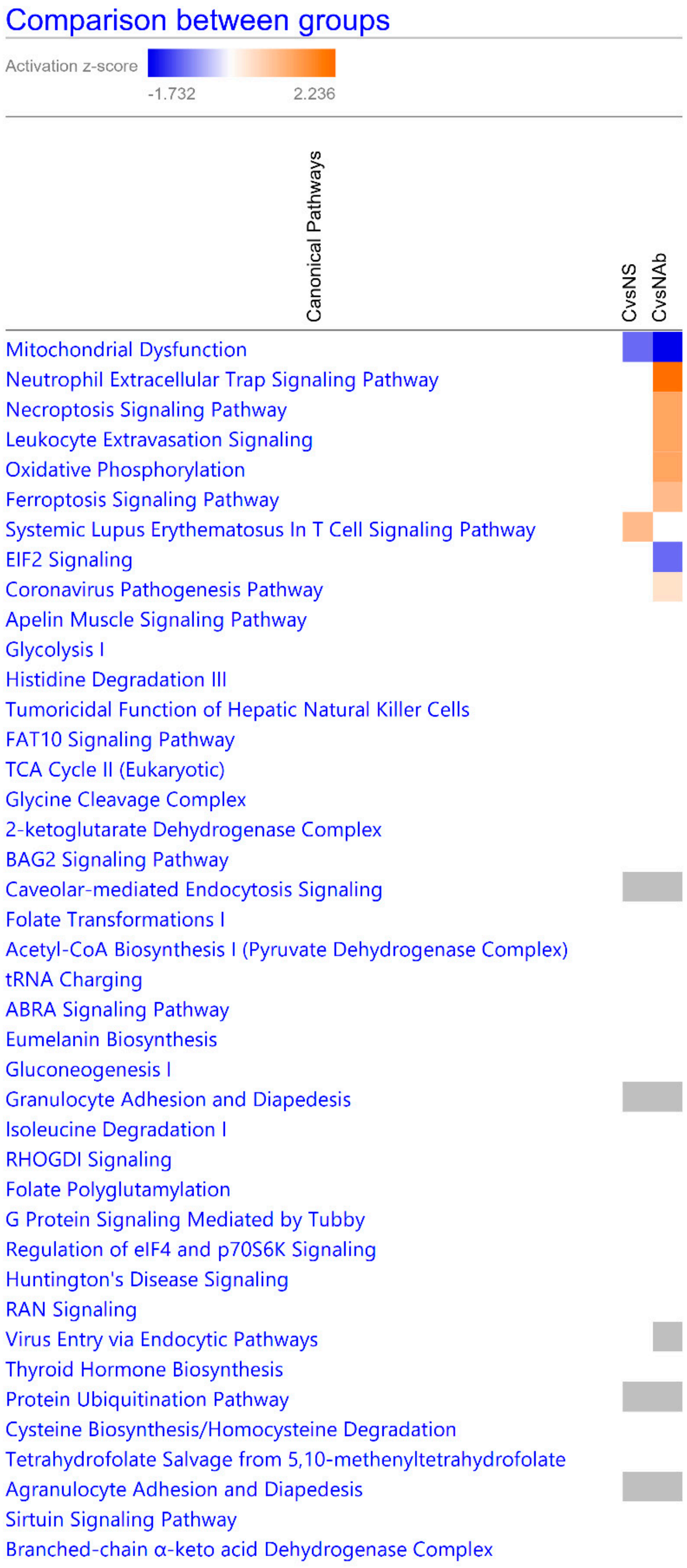

Figure S6: Canonical pathways of iron nanoparticles vs. no treatment **(A)**, NP-Ab vs. no treatment **(B)**, and comparison between groups **(C)**.

A

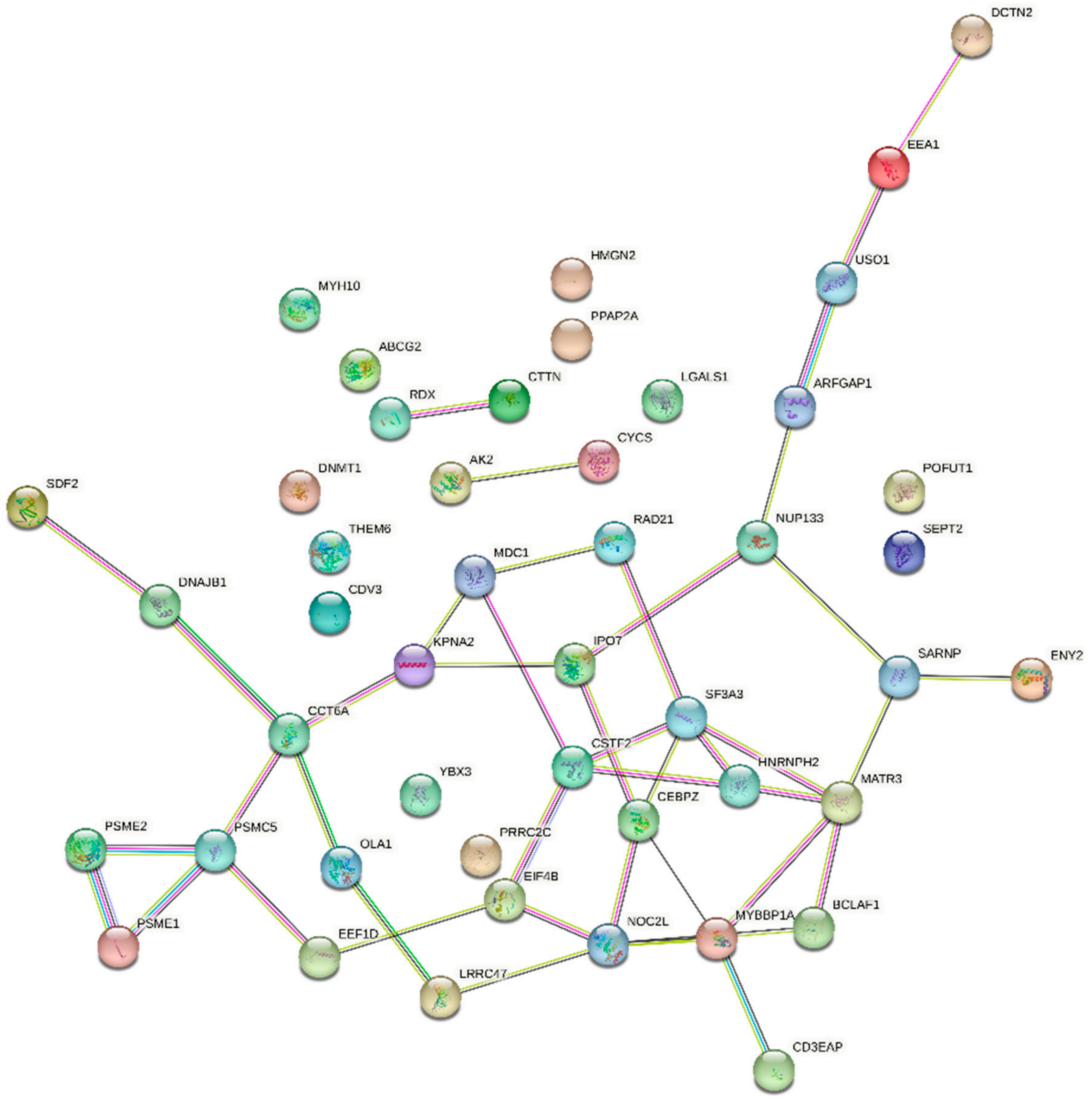
DCTN2
EEA1
USO1
HMGN2
MYH10
PPAP2A
ABCG2
RDX
CTTN
LGALS1
ARFGAP1
CYCS
DNMT1
AK2
POFUT1
SDF2
THEM6
RAD21
NUP133
SEPT2
MDC1
DNAJB1
CDV3
KPNA2
IPO7
SARNP
ENY2
SF3A3
CCT6A
CSTF2
HNRNPH2
YBX3
MATR3
CEBPZ
PSME2
PSMC5
PRRC2C
OLA1
EIF4B
BCLAF1
MYBBP1A
PSME1
NOC2L
EEF1D
LRRC47
CD3EAP

B

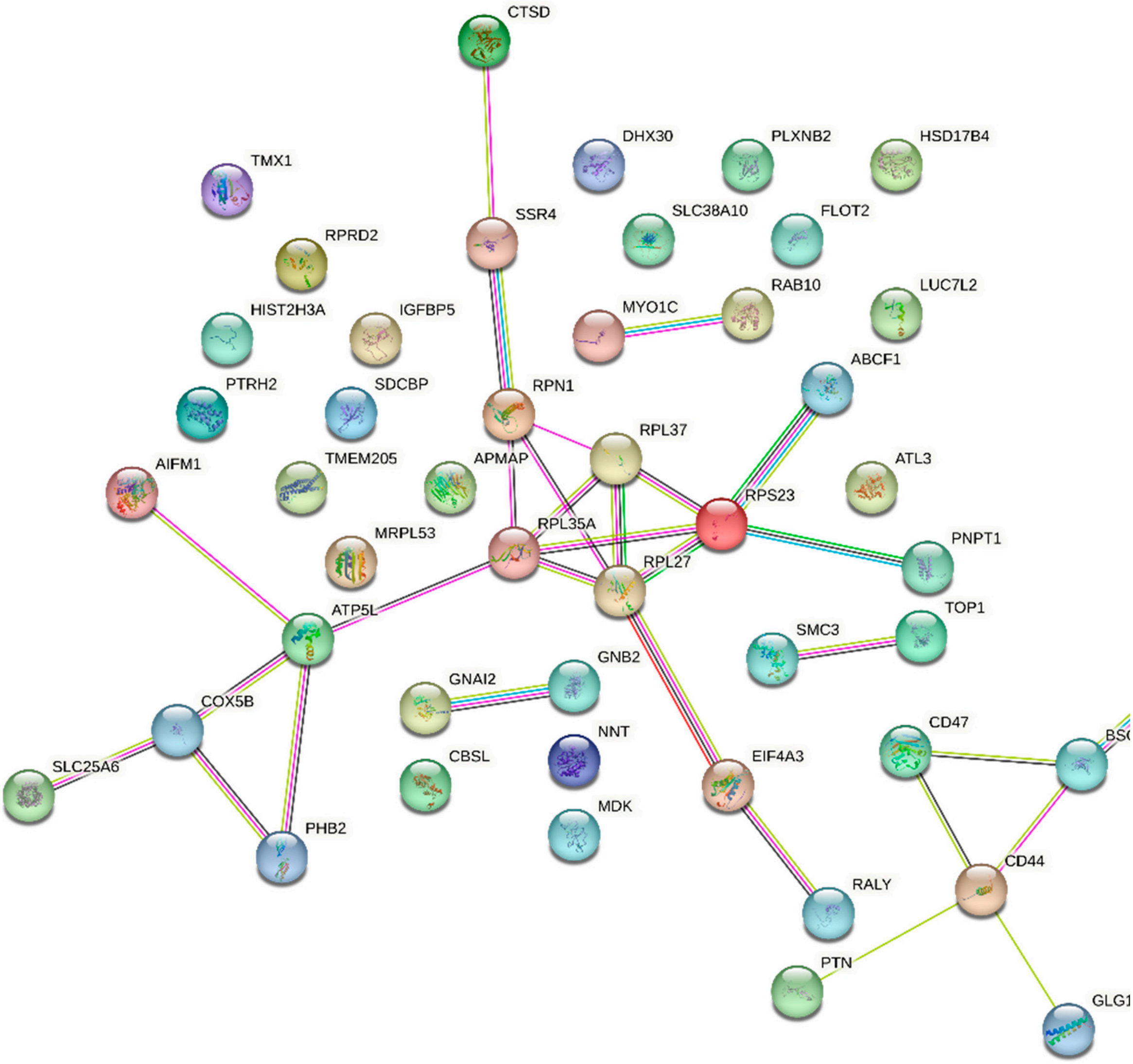
CTSD
TMX1
DHX30
PLXNB2
HSD17B4
SSR4
SLC38A10
FLOT2
RPRD2
HIST2H3A
IGFBP5
MYO1C
RAB10
LUC7L2
PTRH2
SDCBP
RPN1
ABCF1
AIFM1
TMEM205
APMAP
RPL37
ATL3
RPS23
RPL35A
MRPL53
PNPT1
RPL27
ATP5L
TOP1
SMC3
GNB2
GNAI2
COX5B
NNT
CD47
SLC25A6
CBSL
EIF4A3
MDK
PHB2
CD44
RALY
PTN
GLG1

C

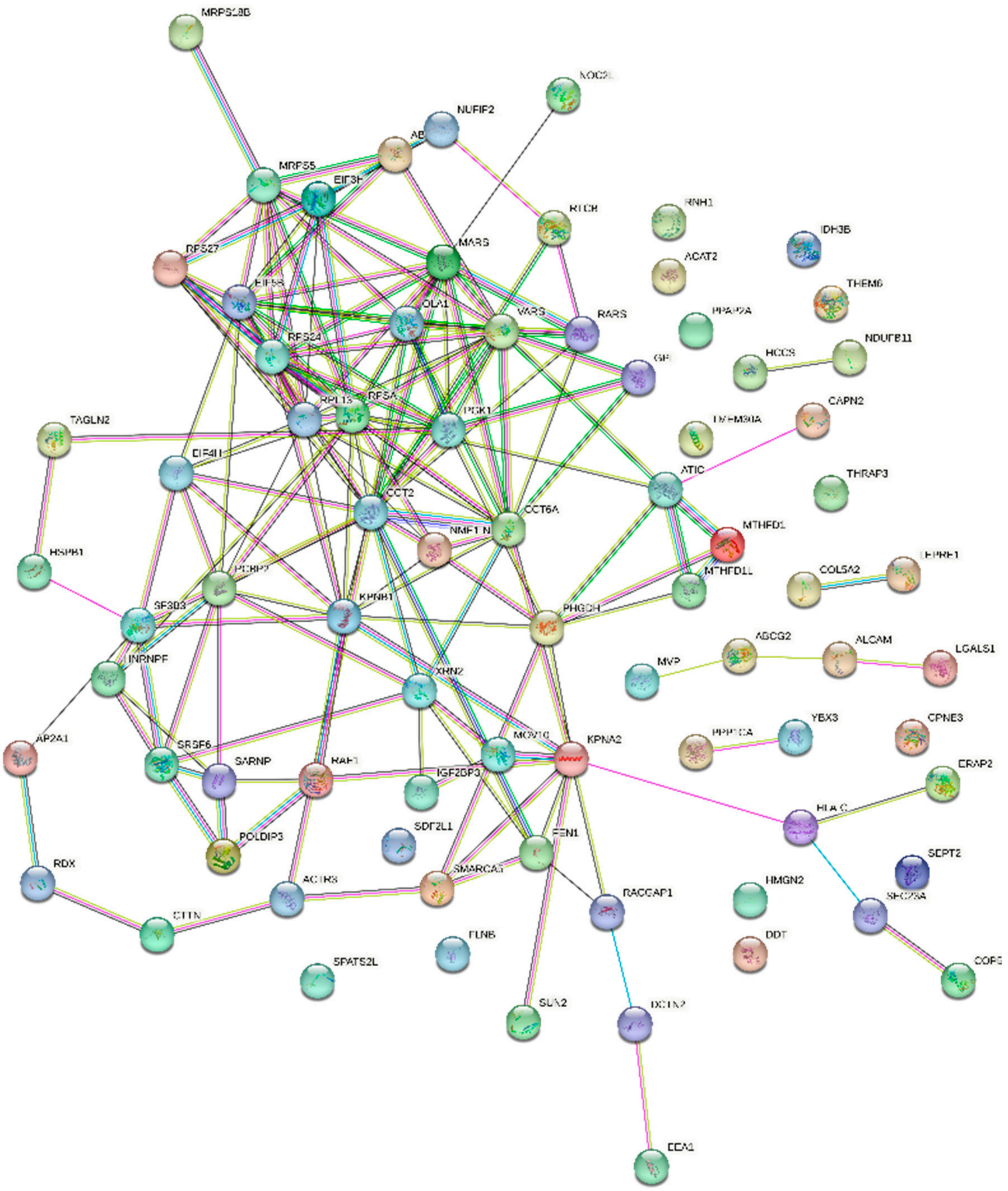
MRPS18B
NUFIP2
MRPS55
EIF3H
RPS27
MARS
EIF5B
OLA1
VARS
RARS
RPS24
RPL13
RPSA
PGK1
TAGLN2
EIF4H
CCT2
CCT6A
HSPB1
PCBP2
SF3B3
KPNB1
PHGDH
HNRNPF
XRN2
AP2A1
SRSF6
SARNP
RAE1
IGF2BP3
MOV10
KPNA2
POLDIP3
SDF2L1
FEN1
RDX
ACTR3
SMARCA5
RACGAP1
CTTN
FLNB
SPATS2L
SUN2
EEA1
RNH1
IDH3B
ACAT2
THEM6
PPAP2A
HCCS
NDUFB11
CAPN2
TMEM30A
ATIC
THRAP3
MTHFD1
MTHFD1L
COL5A2
LEPRE1
MVP
ABCG2
ALCAM
LGALS1
YBX3
CPNE3
ERAP2
HLA-C
SEPT2
HMGN2
SEC23A
DDT

D

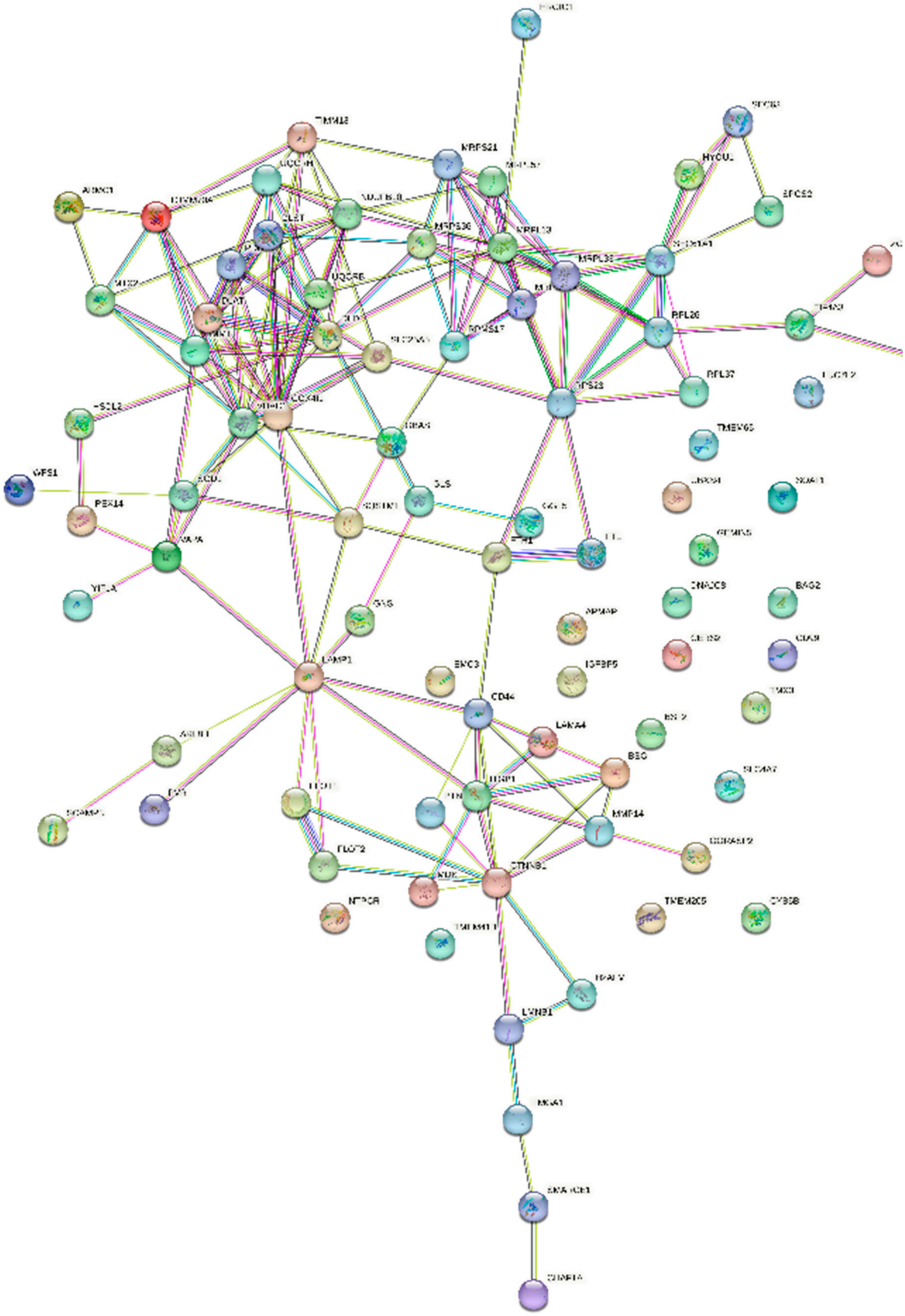

TIMM13
MRPS21
HYOU1
MRPS36
MRPL38
RPL26
RPL37
RPS23
TMEM65
UQCRB
DLAT
DLST
COX4I1
HSDL2
WFS1
PEX14
LAMP1
CD44
LAMA4
BSG
MMP14
CTNNB1
FLOT2
IGFBP5
APMAP
BAG2
TMEM205
CYB5B
LMNB1

Figure S7: STRING diagrams of iron nanoparticles vs. no treatment **(A, B)**, NP-Ab vs. no treatment **(C, D)**, down **(A, C)** and up **(B, D)** regulated proteins.

Table S2: List of significantly differentially expressed proteins in iron nanoparticles vs. no treatment.

Protein names	Gene	T-test (p-value)	log_2(Fold change)
GDP-fucose protein O-fucosyltransferase 1	POFUT1	0.0006	-1.9038
ATP-binding cassette sub-family G member 2	ABCG2	0.0007	-3.9147
Leukocyte surface antigen CD47	CD47	0.0010	2.5729
Protein CDV3 homolog	CDV3	0.0010	-1.2559
Plexin-B2	PLXNB2	0.0013	1.5739
RNA-binding protein Raly	RALY	0.0027	0.3115
Putative sodium-coupled neutral amino acid transporter 10	SLC38A10	0.0043	1.3994
Cytochrome c oxidase subunit 5B, mitochondrial	COX5B	0.0045	4.8150
ATP-binding cassette sub-family F member 1	ABCF1	0.0050	0.3956
Eukaryotic initiation factor 4A-III	EIF4A3	0.0050	2.3533
Atlastin-3	ATL3	0.0052	0.7937
Lipid phosphate phosphohydrolase 1	PPAP2A	0.0053	-0.5620
Large neutral amino acids transporter small subunit 1	SLC7A5	0.0057	0.7714
Adipocyte plasma membrane-associated protein	APMAP	0.0059	0.3165
Midkine	MDK	0.0066	2.9811
Double-strand-break repair protein rad21 homolog	RAD21	0.0072	-0.4798
Myosin-10	MYH10	0.0077	-0.7161
Prohibitin-2	PHB2	0.0079	0.6104
39S ribosomal protein L53, mitochondrial	MRPL53	0.0095	2.0910
Proteasome activator complex subunit 2	PSME2	0.0096	-2.1093
Transmembrane protein 205	TMEM205	0.0105	1.8023
SAP domain-containing ribonucleoprotein	CIP29	0.0113	-1.9063
Peptidyl-tRNA hydrolase 2, mitochondrial	PTRH2	0.0113	0.7309
CCAAT/enhancer-binding protein zeta	CEBPZ	0.0120	-1.0376
Heterogeneous nuclear ribonucleoprotein H2	HNRNPH2	0.0124	-0.4662
Non-histone chromosomal protein HMG-17	HMGN2	0.0139	-0.8954

Regulation of nuclear pre-mRNA domain-containing protein 2	RPRD2	0.0143	0.7157
Guanine nucleotide-binding protein G(I)/G(S)/G(T) subunit beta-2	GNB2	0.0143	0.4417
Src substrate cortactin	CTTN	0.0147	-0.4255
60S ribosomal protein L37	RPL37	0.0153	1.9032
Transcription and mRNA export factor ENY2	ENY2	0.0154	-0.5103
Guanine nucleotide-binding protein G(i) subunit alpha-2	GNAI2	0.0159	0.6008
Proteasome activator complex subunit 1	PSME1	0.0162	-1.4754
Protein THEM6	THEM6	0.0164	-1.3676
Insulin-like growth factor-binding protein 5	IGFBP5	0.0172	1.6481
Stromal cell-derived factor 2	SDF2	0.0176	-1.1884
Splicing factor 3A subunit 3	SF3A3	0.0181	-1.1735
NAD(P) transhydrogenase, mitochondrial	NNT	0.0190	0.6090
Myb-binding protein 1A	MYBBP1A	0.0195	-0.9194
Adenylate kinase 2, mitochondrial	AK2	0.0195	-0.7063
Nucleolar complex protein 2 homolog	NOC2L	0.0201	-0.5056
Mediator of DNA damage checkpoint protein 1	MDC1	0.0209	-0.5937
ADP/ATP translocase 3	SLC25A6	0.0218	0.5081
Putative ATP-dependent RNA helicase DHX30	DHX30	0.0228	1.1354
Septin-2	SEPT2	0.0247	-0.6835
DNA (cytosine-5)-methyltransferase 1	DNMT1	0.0251	-0.6731
Apoptosis-inducing factor 1, mitochondrial	AIFM1	0.0252	0.2921
Nuclear pore complex protein Nup133	NUP133	0.0252	-1.1591
galectin 1	LGALS1	0.0254	-2.3125
Peroxisomal multifunctional enzyme type 2	HSD17B4	0.0261	0.5641
Leucine-rich repeat-containing protein 47	LRRC47	0.0267	-1.2543
Translocon-associated protein subunit delta	SSR4	0.0268	0.3816
ATP synthase subunit g, mitochondrial	ATP5L	0.0273	0.5592
Elongation factor 1-delta	EEF1D	0.0279	-0.3744
ADP-ribosylation factor GTPase-activating protein 1	ARFGAP1	0.0286	-1.3690

Early endosome antigen 1	EEA1	0.0300	-1.3751
Protein PRRC2C	PRRC2C	0.0303	-0.8528
Putative RNA-binding protein Luc7-like 2	LUC7L2	0.0307	0.8823
Pleiotrophin	PTN	0.0308	1.7302
Importin-7	IPO7	0.0309	-1.3591
Thioredoxin-related transmembrane protein 1	TMX1	0.0313	0.7077
Flotillin-2	FLOT2	0.0329	0.6724
DNA-directed RNA polymerase I subunit RPA34	CD3EAP	0.0339	-0.5885
Dolichyl-diphosphooligosaccharide--protein glycosyltransferase subunit 1	RPN1	0.0348	0.4404
Ras-related protein Rab-10	RAB10	0.0348	0.5369
Syntenin-1	SDCBP	0.0348	1.6728
Cytochrome c	CYCS	0.0364	-1.5270
Importin subunit alpha	KPNA2	0.0368	-0.4933
60S ribosomal protein L35a	RPL35A	0.0373	0.5335
Bcl-2-associated transcription factor 1	BCLAF1	0.0374	-0.4765
Eukaryotic translation initiation factor 4B	EIF4B	0.0378	-2.0060
CD44 antigen	CD44	0.0392	0.3942
Unconventional myosin-Ic	MYO1C	0.0401	0.3105
26S protease regulatory subunit 8	PSMC5	0.0402	-1.1658
Polyribonucleotide nucleotidyltransferase 1, mitochondrial	PNPT1	0.0407	0.4901
Obg-like ATPase 1	OLA1	0.0407	-1.1111
General vesicular transport factor p115	USO1	0.0412	-0.7585
Dynactin subunit 2	DCTN2	0.0428	-0.8899
Cleavage stimulation factor subunit 2	CSTF2	0.0440	-0.8107
Golgi apparatus protein 1	GLG1	0.0443	0.4408
40S ribosomal protein S23	RPS23	0.0450	0.6056
Histone H3.2	HIST2H3A	0.0450	0.3266
60S ribosomal protein L27	RPL27	0.0450	0.3076
T-complex protein 1 subunit zeta	CCT6A	0.0458	-1.0169

DNA topoisomerase 1	TOP1	0.0462	0.3740
Matrin-3	MATR3	0.0463	-0.3351
Cystathionine beta-synthase	CBS	0.0470	1.4284
DnaJ homolog subfamily B member 1	DNAJB1	0.0471	-0.7371
Y-box-binding protein 3	CSDA	0.0475	-0.3591
Basigin	EMMPRIN	0.0476	0.2804
Cathepsin D	CTSD	0.0488	0.2758
Structural maintenance of chromosomes protein	SMC3	0.0495	0.3513
Radixin	RDX	0.0498	-0.8543

Table S3: List of significantly differentially expressed proteins in NP-Ab vs. no treatment.

Protein names	Gene	T-test (p-value)	log_2(Fold change)
28S ribosomal protein S17, mitochondrial	MRPS17	0.0001	3.4772
ATP-binding cassette sub-family G member 2	ABCG2	0.0001	-4.0280
Eukaryotic initiation factor 4A-III	EIF4A3	0.0002	2.5383
Poliovirus receptor	PVR	0.0002	1.0296
Midkine	MDK	0.0003	3.4510
SAP domain-containing ribonucleoprotein	CIP29	0.0003	-1.5195
Superoxide dismutase [Cu-Zn]	SOD1	0.0004	2.3078
Protein THEM6	THEM6	0.0004	-1.9948
galectin 1	LGALS1	0.0005	-2.5652
Peroxisomal membrane protein PEX14	PEX14	0.0005	2.2424
Lysosome-associated membrane glycoprotein 1	LAMP1	0.0005	3.6663
Pleiotrophin	PTN	0.0006	1.8087
Adipocyte plasma membrane-associated protein	APMAP	0.0006	0.3352
Transmembrane protein 41B	TMEM41B	0.0008	2.4630
Transmembrane protein 205	TMEM205	0.0008	1.8339
BAG family molecular chaperone regulator 2	BAG2	0.0011	0.4071
Mitochondrial import inner membrane translocase subunit Tim13	TIMM13	0.0011	3.2074
Filamin-B	FLNB	0.0012	-0.5015
Ferritin	FTH1	0.0012	1.2108
Stromal cell-derived factor 2-like protein 1	SDF2L1	0.0013	-1.8811
High mobility group protein HMG-I/HMG-Y	HMGA1	0.0016	0.8241
28S ribosomal protein S18b, mitochondrial	MRPS18B	0.0017	-2.9979

mRNA export factor	RAE1	0.0019	-1.9188
Cytochrome c-type heme lyase	HCCS	0.0021	-1.7306
Poly(rC)-binding protein 2	PCBP2	0.0028	-0.6162
Flap endonuclease 1	FEN1	0.0029	-1.5829
SWI/SNF-related matrix-associated actin-dependent regulator of chromatin subfamily A member 5	SMARCA5	0.0030	-0.7712
28S ribosomal protein S21, mitochondrial	MRPS21	0.0032	1.5755
ADP-ribosylation factor-like protein 8B	ARL8B	0.0033	0.8229
Gamma-glutamyltransferase 5	GGT5	0.0034	2.4295
Insulin-like growth factor-binding protein 5	IGFBP5	0.0036	1.4291
Translocation protein SEC63 homolog	SEC63	0.0039	0.7977
28S ribosomal protein S5, mitochondrial	MRPS5	0.0041	-1.9747
39S ribosomal protein L33, mitochondrial	MRPL33	0.0045	1.5243
Valine--tRNA ligase	VARS	0.0052	-1.8495
Protein disulfide-isomerase TMX3	TMX3	0.0053	2.6733
Nuclear fragile X mental retardation-interacting protein 2	NUFIP2	0.0054	-0.3719
Eukaryotic translation initiation factor 3 subunit H	EIF3H	0.0054	-1.2525
SWI/SNF-related matrix-associated actin-dependent regulator of chromatin subfamily E member 1	SMARCE1	0.0057	1.3343
Endoplasmic reticulum-Golgi intermediate compartment protein 1	ERGIC1	0.0058	1.8188
C-1-tetrahydrofolate synthase, cytoplasmic	MTHFD1	0.0058	-2.2323
ATP-binding cassette sub-family F member 1	ABCF1	0.0059	-1.9886
Anion exchange protein	SLC4A7	0.0061	0.7393
Lipid phosphate phosphohydrolase 1	PPAP2A	0.0066	-1.4439
Armadillo repeat-containing protein 1	ARMC1	0.0068	1.9002

Coatomer subunit beta	COPB2	0.0070	-2.3003
T-complex protein 1 subunit beta	CCT2	0.0073	-0.8644
60S ribosomal protein L13	RPL13	0.0075	-0.3719
Ceramide synthase 2	CERS2	0.0084	2.2992
Splicing factor 3B subunit 3	SF3B3	0.0090	-1.5410
Isocitrate dehydrogenase [NAD] subunit, mitochondrial	IDH3B	0.0090	-1.6173
Cancer-related nucleoside-triphosphatase	NTPCR	0.0093	1.3784
Methionine--tRNA ligase, cytoplasmic	MARS	0.0098	-0.6055
Secretory carrier-associated membrane protein 3	SCAMP3	0.0102	0.6350
Early endosome antigen 1	EEA1	0.0102	-1.6817
Flotillin-2	FLOT2	0.0107	0.6411
Flotillin-1	FLOT1	0.0107	1.3345
40S ribosomal protein S27	RPS27	0.0111	-0.7153
Rac GTPase-activating protein 1	RACGAP1	0.0114	-1.0788
Y-box-binding protein 3	CSDA	0.0118	-0.5607
40S ribosomal protein S23	RPS23	0.0122	0.8086
SPATS2-like protein	DNAPTP6	0.0123	-0.4884
Thyroid hormone receptor-associated protein 3	THRAP3	0.0126	-0.4759
NADH dehydrogenase [ubiquinone] 1 beta subcomplex subunit 11, mitochondrial	NDUFB11	0.0127	-3.3131
Eukaryotic translation initiation factor 4H	EIF4H	0.0127	-2.3031
Heterogeneous nuclear ribonucleoprotein F	HNRPF	0.0134	-0.5895
Protein transport protein Sec23A	SEC23A	0.0136	-1.6195
D-3-phosphoglycerate dehydrogenase	PHGDH	0.0140	-0.6366
Protein NipSnap homolog 2	GBAS	0.0147	1.0788

Putative RNA-binding protein Luc7-like 2	LUC7L2	0.0152	1.1515
Laminin subunit alpha-4	LAMA4	0.0154	0.7832
Lamin-B1	LMNB1	0.0161	0.2827
Golgi reassembly-stacking protein 2	GORASP2	0.0164	1.7081
Prolyl 3-hydroxylase 1	LEPRE1	0.0167	-1.2940
Dihydrolipoyl dehydrogenase	DLD	0.0167	0.3026
Calpain-2 catalytic subunit	CAPN2	0.0171	-1.3587
Collagen alpha-2(V) chain	COL5A2	0.0171	-1.4330
60S ribosomal protein L26	RPL26	0.0178	0.6681
Actin-related protein 3	ACTR3	0.0179	-0.4475
Polymerase delta-interacting protein 3	POLDIP3	0.0179	-0.9405
Nucleolar complex protein 2 homolog	NOC2L	0.0185	-0.9860
Septin-2	SEPT2	0.0185	-0.3581
28S ribosomal protein S18a, mitochondrial	MRPS18A	0.0188	0.6309
UBX domain-containing protein 4	UBXN4	0.0189	0.7451
Transmembrane protein 65	TMEM65	0.0198	1.2945
Copine-3	CPNE3	0.0199	-1.5767
Cytochrome c oxidase subunit 4 isoform 1, mitochondrial	COX4I1	0.0200	0.7576
glutaminase	GLS	0.0202	0.9063
N-acetylglucosamine-6-sulfatase	GNS	0.0204	2.5720
Histone H2A.V	H2AFV	0.0207	0.6423
Cell cycle control protein 50A	TMEM30A	0.0209	-1.0582
28S ribosomal protein S36, mitochondrial	MRPS36	0.0209	0.4672
Transgelin-2	TAGLN2	0.0211	-1.2663

Putative helicase MOV-10	MOV10	0.0213	-0.9536
Src substrate cortactin	CTTN	0.0214	-0.3318
Importin subunit alpha	KPNA2	0.0215	-0.3920
Cytochrome b-c1 complex subunit 7	UQCRB	0.0216	0.5363
Sequestosome-1	SQSTM1	0.0216	0.4922
Matrix metalloproteinase-14	MMP14	0.0220	1.0464
Acetyl-CoA acetyltransferase, cytosolic	ACAT2	0.0220	-1.4965
SUN domain-containing protein 2	SUN2	0.0223	-1.4274
Major vault protein	MVP	0.0224	-0.3699
MICOS complex subunit MIC60	IMMT	0.0224	0.3500
Non-histone chromosomal protein HMG-17	HMGN2	0.0232	-0.6104
Obg-like ATPase 1	OLA1	0.0239	-1.5918
Glucose-6-phosphate isomerase	GPI	0.0243	-2.1884
40S ribosomal protein S24	RPS24	0.0266	-0.5799
Gem-associated protein 5	GEMIN5	0.0268	0.8453
Pyruvate dehydrogenase E1 component subunit alpha	PDHA1	0.0268	0.3149
Serine/threonine-protein phosphatase PP1-alpha catalytic subunit	PPP1CA	0.0271	-0.4546
Wolframin	WFS1	0.0278	1.4713
Nucleoside diphosphate kinase	NME1-NME2	0.0279	-0.6432
D-dopachrome decarboxylase	DDT	0.0281	-1.1471
Dihydrolipoyllysine-residue succinyltransferase component of 2-oxoglutarate dehydrogenase complex, mitochondrial	DLST	0.0294	0.4810
Protein YIF1A	YIF1A	0.0296	1.6141
39S ribosomal protein L13, mitochondrial	MRPL13	0.0303	0.9888

Hypoxia up-regulated protein 1	HYOU1	0.0305	0.3840
Zinc finger CCCH domain-containing protein 14	ZC3H14	0.0305	1.0385
NADH dehydrogenase [ubiquinone] 1 beta subcomplex subunit 10	NDUFB10	0.0307	0.5275
Voltage-dependent anion-selective channel protein 1	VDAC1	0.0308	0.4137
Mitochondrial import receptor subunit TOM70	TOMM70A	0.0312	0.4007
Insulin-like growth factor 2 mRNA-binding protein 3	IGF2BP3	0.0315	-0.3846
Metaxin-2	MTX2	0.0324	1.2495
Basigin	EMMPRIN	0.0331	0.4975
Arginine--tRNA ligase, cytoplasmic	RARS	0.0331	-0.8051
Heat shock protein beta-1	HSPB1	0.0337	-0.6896
60S ribosomal protein L37	RPL37	0.0339	1.8160
Heterogeneous nuclear ribonucleoprotein H2	HNRNPH2	0.0342	0.4639
Vesicle-associated membrane protein-associated protein A	VAPA	0.0342	0.3434
Eukaryotic translation initiation factor 5B	EIF5B	0.0343	-0.4981
T-complex protein 1 subunit zeta	CCT6A	0.0360	-0.6830
major histocompatibility complex, class I, C	HLA-C	0.0363	-0.4223
Importin subunit beta-1	KPNB1	0.0371	-0.4539
ADP/ATP translocase 2	SLC25A5	0.0383	0.4003
Ferritin light chain	FTL	0.0391	0.7215
Bone marrow stromal antigen 2	BST2	0.0391	1.6869
Protein transport protein Sec61 subunit alpha isoform 1	SEC61A1	0.0397	0.9844
DnaJ homolog subfamily C member 8	DNAJC8	0.0400	1.4623
CD44 antigen	CD44	0.0402	0.2670
AP-2 complex subunit alpha-1	AP2A1	0.0408	-1.0962

Cytochrome b5 type B	CYB5B	0.0410	0.6904
Cytochrome b-c1 complex subunit 6, mitochondrial	UQCRH	0.0414	0.2658
Integrin beta	ITGB1	0.0418	0.3131
tRNA-splicing ligase RtcB homolog	RTCB	0.0418	-1.3176
ER membrane protein complex subunit 3	EMC3	0.0421	0.4650
Endoplasmic reticulum aminopeptidase 2	ERAP2	0.0424	-0.5146
Ribosomal protein 63, mitochondrial	MRP63	0.0428	1.1748
Signal peptidase complex subunit 2	SPCS2	0.0430	0.4859
Catenin beta-1	CTNNB1	0.0432	0.3634
Radixin	RDX	0.0432	-0.3131
Bifunctional purine biosynthesis protein PURH	ATIC	0.0434	-1.1070
Chromatin assembly factor 1 subunit A	CHAF1A	0.0435	1.0982
CD99 antigen	CD99	0.0445	2.5502
Sterol O-acyltransferase 1	SOAT1	0.0445	0.6573
Mitochondrial import inner membrane translocase subunit TIM44	hTIM44	0.0447	0.4991
Ribonuclease inhibitor	RNH1	0.0466	-1.5893
40S ribosomal protein SA	RPSA	0.0467	-0.5067
Serine/arginine-rich splicing factor 6	SRSF6	0.0469	-1.2163
Hydroxysteroid dehydrogenase-like protein 2	HSDL2	0.0472	0.3804
Monofunctional C1-tetrahydrofolate synthase, mitochondrial	MTHFD1L	0.0478	-0.5552
CD166 antigen	ALCAM	0.0483	-0.6400
Dihydrolipoyllysine-residue acetyltransferase component of pyruvate dehydrogenase complex, mitochondrial	DLAT	0.0485	0.6243
5-3 exoribonuclease 2	XRN2	0.0496	-0.4001

Dynactin subunit 2	DCTN2	0.0497	-0.8707
Phosphoglycerate kinase	PGK1	0.0498	-0.6840

Table S4: List of overlapped DEPs across different nanoparticle treatment (iron nanoparticles and NP-Ab).

Protein names	Gene	Iron nanoparticle vs. control		NP-Ab vs. control	
		T-test (p-value)	log_2(Fold change)	T-test (p-value)	log_2(Fold change)
ATP-binding cassette sub-family G member 2	ABCG2	0.0007	-3.9147	0.0001	-4.0280
T-complex protein 1 subunit zeta	CCT6A	0.0458	-1.0169	0.0360	-0.6830
SAP domain-containing ribonucleoprotein	CIP29	0.0113	-1.9063	0.0003	-1.5195
Y-box-binding protein 3	CSDA	0.0475	-0.3591	0.0118	-0.5607
Src substrate cortactin	CTTN	0.0147	-0.4255	0.0214	-0.3318
Dynactin subunit 2	DCTN2	0.0428	-0.8899	0.0497	-0.8707
Early endosome antigen 1	EEA1	0.0300	-1.3751	0.0102	-1.6817
Non-histone chromosomal protein HMG-17	HMGN2	0.0139	-0.8954	0.0232	-0.6104
Importin subunit alpha	KPNA2	0.0368	-0.4933	0.0215	-0.3920
galectin 1	LGALS1	0.0254	-2.3125	0.0005	-2.5652
Nucleolar complex protein 2 homolog	NOC2L	0.0201	-0.5056	0.0185	-0.9860
Obg-like ATPase 1	OLA1	0.0407	-1.1111	0.0239	-1.5918
Lipid phosphate phosphohydrolase 1	PPAP2A	0.0053	-0.5620	0.0066	-1.4439
Radixin	RDX	0.0498	-0.8543	0.0432	-0.3131
Septin-2	SEPT2	0.0247	-0.6835	0.0185	-0.3581
Protein THEM6	THEM6	0.0164	-1.3676	0.0004	-1.9948
Adipocyte plasma membrane-associated protein	APMAP	0.0059	0.3165	0.0006	0.3352
CD44 antigen	CD44	0.0392	0.3942	0.0402	0.2670
Eukaryotic initiation factor 4A-III	EIF4A3	0.0050	2.3533	0.0002	2.5383
Flotillin-2	FLOT2	0.0329	0.6724	0.0107	0.6411

Insulin-like growth factor-binding protein 5	IGFBP5	0.0172	1.6481	0.0036	1.4291
Putative RNA-binding protein Luc7-like 2	LUC7L2	0.0307	0.8823	0.0152	1.1515
Midkine	MDK	0.0066	2.9811	0.0003	3.4510
Pleiotrophin	PTN	0.0308	1.7302	0.0006	1.8087
60S ribosomal protein L37	RPL37	0.0153	1.9032	0.0339	1.8160
40S ribosomal protein S23	RPS23	0.0450	0.6056	0.0122	0.8086
Transmembrane protein 205	TMEM205	0.0105	1.8023	0.0008	1.8339
Basigin	EMMPRIN	0.0476	0.2804	0.0331	0.4975

Table S5: Gene ontology enrichment analysis of DEPs (iron nanoparticles vs. control).

Term		Count	%	Genes	Fold Enrichment	p-value
**Biological Process**						
GO:0007062	sister chromatid cohesion	3	3.5294	RAD21, SMC3, PHB2	27.5399	0.0052
GO:0002181	cytoplasmic translation	4	4.7059	RPL35A, RPL27, RPL37, RPS23	10.6080	0.0062
GO:0006606	protein import into nucleus	4	4.7059	NUP133, KPNA2, PHB2, IPO7	9.1800	0.0092
GO:0006915	apoptotic process	8	9.4118	BCLAF1, LGALS1, AIFM1, PTRH2, RAD21, CYCS, NOC2L, SLC25A6	3.1457	0.0128
GO:0006412	translation	5	5.8824	RPL35A, RPL27, RPL37, RPS23, ABCF1	5.3515	0.0138
GO:0034089	establishment of meiotic sister chromatid cohesion	2	2.3529	RAD21, SMC3	119.3395	0.0165
GO:1904707	positive regulation of vascular smooth muscle cell proliferation	3	3.5294	DNMT1, IGFBP5, GNAI2	12.7864	0.0225
GO:0030335	positive regulation of cell migration	5	5.8824	SDCBP, MYO1C, MDK, RDX, GNAI2	4.5204	0.0240
GO:0006406	mRNA export from nucleus	3	3.5294	ENY2, NUP133, EIF4A3	12.3455	0.0241
GO:0008360	regulation of cell shape	4	4.7059	RDX, PLXNB2, PTN, MYH10	6.1995	0.0261
GO:0034087	establishment of mitotic sister chromatid cohesion	2	2.3529	RAD21, SMC3	68.1940	0.0286
GO:2000210	positive regulation of anoikis	2	2.3529	MYBBP1A, PTRH2	68.1940	0.0286
GO:1905460	negative regulation of vascular associated smooth muscle cell apoptotic process	2	2.3529	SLC7A5, DNMT1	68.1940	0.0286
GO:0002232	leukocyte chemotaxis involved in inflammatory response	2	2.3529	MDK, PTN	59.6698	0.0326

GO:0006930	substrate-dependent cell migration, cell extension	2	2.3529	SDCBP, CTTN	53.0398	0.0366
GO:2000347	positive regulation of hepatocyte proliferation	2	2.3529	MDK, PTN	43.3962	0.0446
GO:0045727	positive regulation of translation	3	3.5294	EIF4A3, PLXNB2, ABCF1	8.5243	0.0474
GO:0032330	regulation of chondrocyte differentiation	2	2.3529	MDK, GLG1	39.7798	0.0486
GO:0043065	positive regulation of apoptotic process	5	5.8824	BCLAF1, LGALS1, AIFM1, PTN, CTSD	3.5946	0.0489
**Molecular Function**						
GO:0003723	RNA binding	30	35.2941	DNMT1, CSTF2, USO1, RPN1, EIF4A3, NOC2L, DHX30, BCLAF1, LGALS1, RALY, RPL37, HMGN2, KPNA2, EIF4B, ABCF1, SF3A3, LRRC47, PNPT1, RDX, RPL35A, PRRC2C, CCT6A, MYBBP1A, RPL27, CEBPZ, HNRNPH2, MATR3, TOP1, LUC7L2, RPS23	4.7026	<0.0001
GO:0005515	protein binding	71	83.5294	MDC1, EIF4A3, OLA1, SMC3, NOC2L, IPO7, PHB2, EEA1, LGALS1, AIFM1, APMAP, MDK, FLOT2, HMGN2, KPNA2, CTSD, LRRC47, IGFBP5, NUP133, ARFGAP1, MRPL53, SLC7A5, CTTN, TMX1, EEF1D, PTRH2, PSME1, PSME2, RPL27, MATR3, HNRNPH2, CD47, TOP1, LUC7L2, CD44, SLC25A6, ABCG2, ENY2, DNMT1, DCTN2, ATL3, USO1, RPN1, CSTF2, AK2, PTN, COX5B, GNAI2, DNAJB1, SDCBP, DHX30, BCLAF1, CBS, TMEM205, RAD21, RALY, MYH10, EIF4B, ABCF1, SF3A3, PNPT1, RDX, RPL35A, RAB10, CCT6A, PSMC5, MYO1C, GNB2, CYCS, PLXNB2, RPS23	1.2974	<0.0001
GO:0003682	chromatin binding	8	9.4118	DHX30, DNMT1, ENY2, RAD21, TOP1, SMC3, NOC2L, HMGN2	3.9332	0.0040
GO:0016887	ATPase activity	7	8.2353	CCT6A, DHX30, PSMC5, EIF4A3, OLA1, SMC3, MYH10	4.5596	0.0041
GO:0045296	cadherin binding	6	7.0588	DNAJB1, CTTN, EEF1D, USO1, RDX, OLA1	4.3782	0.0115
GO:0008022	protein C-terminus binding	5	5.8824	SDCBP, MDC1, MYO1C, PRRC2C, PHB2	5.5164	0.0124
GO:0045545	syndecan binding	2	2.3529	SDCBP, PTN	76.8618	0.0254
GO:1904399	heparan sulfate binding	2	2.3529	MDK, PTN	65.8815	0.0296
GO:0035374	chondroitin sulfate binding	2	2.3529	MDK, PTN	46.1171	0.0420
GO:0061133	endopeptidase activator activity	2	2.3529	PSME1, PSME2	41.9246	0.0461

GO:0003735	structural constituent of ribosome	4	4.7059	RPL35A, RPL27, RPL37, RPS23	4.8801	0.0476
**Cellular Component**						
GO:0042788	polysomal ribosome	3	3.5294	PNPT1, RPS23, ABCF1	22.2727	0.0078
GO:0008537	proteasome activator complex	2	2.3529	PSME1, PSME2	163.3333	0.0121
GO:0005743	mitochondrial inner membrane	7	8.2353	MRPL53, AIFM1, NNT, CYCS, COX5B, PHB2, SLC25A6	3.5804	0.0129
GO:0005739	mitochondrion	13	15.2941	ENY2, PNPT1, AK2, COX5B, PHB2, SDCBP, DHX30, MRPL53, AIFM1, NNT, PTRH2, CYCS, SLC25A6	2.1950	0.0133
GO:0016363	nuclear matrix	4	4.7059	RAD21, MATR3, SMC3, PHB2	7.5385	0.0157
GO:0045121	membrane raft	5	5.8824	SDCBP, MYO1C, CTSD, GNAI2, ABCG2	4.8419	0.0192
GO:0045202	synapse	7	8.2353	SDCBP, RPL35A, RPL27, RPL37, ARFGAP1, GNAI2, RPS23	3.2543	0.0197
GO:0005694	chromosome	5	5.8824	MDC1, RAD21, TOP1, SMC3, NOC2L	4.7852	0.0200
GO:0000502	proteasome complex	3	3.5294	PSMC5, PSME1, PSME2	12.8947	0.0222
GO:0098591	external side of apical plasma membrane	2	2.3529	SLC7A5, ABCG2	81.6667	0.0240
GO:0034991	nuclear meiotic cohesin complex	2	2.3529	RAD21, SMC3	81.6667	0.0240
GO:0022625	cytosolic large ribosomal subunit	3	3.5294	RPL35A, RPL27, RPL37	12.2500	0.0245
GO:0030864	cortical actin cytoskeleton	3	3.5294	CTTN, RDX, FLOT2	12.2500	0.0245
GO:0005791	rough endoplasmic reticulum	3	3.5294	RPN1, RPL27, RPS23	11.8548	0.0260
GO:0031982	vesicle	4	4.7059	MYO1C, DCTN2, GNB2, FLOT2	5.6322	0.0334
GO:0005912	adherens junction	4	4.7059	RAB10, SDCBP, RDX, FLOT2	5.5682	0.0343
GO:0005902	microvillus	3	3.5294	MYO1C, RDX, CD44	9.9324	0.0360
GO:0098978	glutamatergic synapse	6	7.0588	EEA1, DNAJB1, EIF4A3, FLOT2, KPNA2, PHB2	3.2379	0.0366
GO:0030496	midbody	4	4.7059	RAD21, RDX, MYH10, GNAI2	5.2973	0.0389

GO:0042564	NLS-dependent protein nuclear import complex	2	2.3529	MYBBP1A, KPNA2	49.0000	0.0396
GO:0005783	endoplasmic reticulum	10	11.7647	CTTN, ATL3, SSR4, EEF1D, POFUT1, USO1, RPN1, PTN, SDF2, RPS23	2.1416	0.0406
GO:0030027	lamellipodium	4	4.7059	CTTN, RDX, FLOT2, MYH10	5.1042	0.0427
GO:0009986	cell surface	7	8.2353	LGALS1, APMAP, PLXNB2, CD47, PTN, PHB2, CD44	2.6713	0.0451
GO:0035145	exon-exon junction complex	2	2.3529	BCLAF1, EIF4A3	40.8333	0.0473

Table S6: Gene ontology enrichment analysis of DEPs (NP-Ab vs. control).

Term		Count	%	Genes	Fold Enrichment	p-value
**Biological Process**						
GO:0006412	translation	17	11.4865	MRPS17, MRPS18B, MRPS18A, MRPS21, RPSA, MRPL13, MRPS5, MRPL33, RPS27, GEMIN5, RPL13, IGF2BP3, RPL26, RPL37, RPS24, RPS23, ABCF1	10.3790	<0.0001
GO:0002181	cytoplasmic translation	7	4.7297	RPS27, RPL13, RPSA, RPL37, RPL26, RPS24, RPS23	10.5893	0.0001
GO:0032543	mitochondrial translation	7	4.7297	MRPS17, MRPS18B, MRPS18A, MRPS21, MRPL13, MRPS5, MRPL33	9.9275	0.0001
GO:0006099	tricarboxylic acid cycle	5	3.3784	PDHA1, MRPS36, IDH3B, DLST, DLAT	20.6285	0.0001
GO:0006103	2-oxoglutarate metabolic process	4	2.7027	MRPS36, IDH3B, DLST, DLD	30.2551	0.0003
GO:0006086	acetyl-CoA biosynthetic process from pyruvate	3	2.0270	PDHA1, DLAT, DLD	68.0739	0.0008
GO:0006446	regulation of translational initiation	4	2.7027	EIF5B, EIF4H, HSPB1, EIF3H	17.5675	0.0015
GO:0006888	ER to Golgi vesicle-mediated transport	6	4.0541	COPB2, SEC23A, VAPA, HYOU1, YIF1A, ERGIC1	6.2358	0.0027
GO:0050821	protein stabilization	7	4.7297	CCT6A, CCT2, BAG2, LAMP1, WFS1, FLOT1, FLOT2	4.3320	0.0056
GO:0006164	purine nucleotide biosynthetic process	3	2.0270	SLC4A7, ATIC, MTHFD1	25.5277	0.0059
GO:0009257	10-formyltetrahydrofolate biosynthetic process	2	1.3514	MTHFD1, MTHFD1L	136.1479	0.0145
GO:0009060	aerobic respiration	4	2.7027	UQCRB, NDUFB10, NDUFB11, UQCRH	7.7799	0.0146
GO:0006364	rRNA processing	5	3.3784	RPS27, XRN2, EIF4A3, RPL26, RPS24	5.1965	0.0156
GO:0017148	negative regulation of translation	4	2.7027	IGFBP5, EIF4A3, IGF2BP3, MRPL13	6.5613	0.0228
GO:0045727	positive regulation of translation	4	2.7027	POLDIP3, EIF4A3, RPL26, ABCF1	6.4832	0.0236

GO:0034976	response to endoplasmic reticulum stress	4	2.7027	GORASP2, WFS1, FLOT1, HYOU1	6.4070	0.0243
GO:0032212	positive regulation of telomere maintenance via telomerase	3	2.0270	CCT6A, CCT2, CTNNB1	12.0130	0.0254
GO:0007405	neuroblast proliferation	3	2.0270	ITGB1, RACGAP1, CTNNB1	11.3457	0.0283
GO:0090114	COPII-coated vesicle budding	2	1.3514	SEC23A, VAPA	54.4592	0.0359
GO:0061732	mitochondrial acetyl-CoA biosynthetic process from pyruvate	2	1.3514	PDHA1, DLD	54.4592	0.0359
GO:0061077	chaperone-mediated protein folding	3	2.0270	CCT6A, CCT2, HSPB1	9.9620	0.0360
GO:0045333	cellular respiration	3	2.0270	UQCRB, COX4I1, UQCRH	9.7248	0.0376
GO:0046718	viral entry into host cell	4	2.7027	ITGB1, LAMP1, RPSA, PVR	5.3391	0.0387
GO:0015031	protein transport	8	5.4054	SEC61A1, SEC23A, MTX2, MVP, TIMM13, YIF1A, SCAMP3, ARL8B	2.4811	0.0422
GO:1901526	positive regulation of macromitophagy	2	1.3514	VDAC1, SLC25A5	45.3826	0.0430
GO:0006880	intracellular sequestering of iron ion	2	1.3514	FTH1, FTL	45.3826	0.0430
GO:0001765	membrane raft assembly	2	1.3514	FLOT1, FLOT2	45.3826	0.0430
GO:2000675	negative regulation of type B pancreatic cell apoptotic process	2	1.3514	WFS1, SRSF6	45.3826	0.0430
GO:0030335	positive regulation of cell migration	6	4.0541	ITGB1, SUN2, MMP14, MDK, RDX, CPNE3	3.0943	0.0444
GO:0032355	response to estradiol	4	2.7027	GGT5, GPI, CTNNB1, PTN	4.8624	0.0487
GO:2000391	positive regulation of neutrophil extravasation	2	1.3514	MDK, CD99	38.8994	0.0500
**Molecular Function**						

GO:0003723	RNA binding	46	31.0811	NUFIP2, POLDIP3, RTCB, EIF4A3, HSPB1, NTPCR, NOC2L, MRPL13, LGALS1, PCBP2, EIF4H, RPL13, FLNB, IGF2BP3, CPNE3, RPL37, HMGN2, KPNA2, ZC3H14, RAE1, ABCF1, SEC63, SMARCE1, EIF5B, RDX, HMGA1, IMMT, MRPS21, RPSA, MRPS5, CCT6A, BST2, MOV10, RPS27, THRAP3, XRN2, GEMIN5, EIF3H, HNRNPH2, RPL26, SRSF6, SLC25A5, RPS24, LUC7L2, KPNB1, RPS23	4.0498	<0.0001
GO:0003735	structural constituent of ribosome	14	9.4595	MRPS17, MRPS18B, MRPS18A, MRPS21, RPSA, MRPL13, MRPS5, MRPL33, RPS27, RPL13, RPL26, RPL37, RPS24, RPS23	9.5931	<0.0001
GO:0005515	protein binding	127	85.8108	RNH1, TMEM41B, RTCB, EIF4A3, NOC2L, PVR, LGALS1, ALCAM, APMAP, LAMP1, FTH1, CAPN2, CPNE3, HMGN2, KPNA2, SEC63, IGFBP5, MRPS18B, HLA-C, RPSA, ARMC1, TMX3, MTHFD1, SOAT1, XRN2, GEMIN5, TAGLN2, VDAC1, RPL26, SRSF6, SQSTM1, KPNB1, ARL8B, FTL, ABCG2, COPB2, NDUFB10, UQCRB, NDUFB11, MRPL13, GNS, ACAT2, UQCRH, BAG2, RACGAP1, PCBP2, PGK1, ZC3H14, CD99, PDHA1, SDF2L1, WFS1, RDX, SMARCA5, HMGA1, CCT6A, BST2, MOV10, RPS27, HYOU1, RPS23, ITGB1, MRPS17, GPI, FEN1, COX4I1, OLA1, HSPB1, AP2A1, HCCS, LMNB1, EEA1, SEC61A1, CHAF1A, MDK, FLOT1, FLOT2, DLAT, UBXN4, ACTR3, CCT2, SUN2, EIF5B, MMP14, CTTN, GORASP2, THRAP3, HNRNPH2, DNAJC8, SLC25A5, DLD, ERGIC1, LUC7L2, CD44, CERS2, SEC23A, SF3B3, DCTN2, NUFIP2, POLDIP3, MVP, TIMM13, DLST, PTN, TMEM65, TMEM205, EMC3, EIF4H, RPL13, FLNB, IGF2BP3, RAE1, ABCF1, CYB5B, SMARCE1, TMEM30A, MTX2, IMMT, PEX14, YIF1A, PPP1CA, SOD1, SLC4A7, SPCS2, VAPA, CTNNB1, EIF3H	1.3034	<0.0001
GO:0045296	cadherin binding	10	6.7568	ITGB1, ATIC, CTTN, VAPA, RDX, EIF4H, OLA1, CTNNB1, TAGLN2, FLNB	4.0983	0.0008
GO:0034604	pyruvate dehydrogenase (NAD+) activity	3	2.0270	PDHA1, DLAT, DLD	64.7534	0.0009
GO:0043022	ribosome binding	5	3.3784	SEC61A1, OLA1, GEMIN5, RPSA, ABCF1	8.9935	0.0023
GO:0031625	ubiquitin protein ligase binding	8	5.4054	CCT2, GPI, BAG2, WFS1, PCBP2, SLC25A5, SQSTM1, SCAMP3	3.3748	0.0096
GO:0003729	mRNA binding	7	4.7297	POLDIP3, PCBP2, EIF4A3, IGF2BP3, SRSF6, MRPL13, LUC7L2	3.7002	0.0116
GO:0008320	protein transmembrane transporter activity	3	2.0270	SEC61A1, PEX14, SEC63	16.1884	0.0144
GO:0004329	formate-tetrahydrofolate ligase activity	2	1.3514	MTHFD1, MTHFD1L	129.5068	0.0153
GO:0043236	laminin binding	3	2.0270	ITGB1, LGALS1, RPSA	14.9431	0.0168
GO:0051082	unfolded protein binding	5	3.3784	CCT6A, CCT2, CHAF1A, HSPB1, HYOU1	4.9430	0.0184

GO:0035255	ionotropic glutamate receptor binding	3	2.0270	FLOT1, FLOT2, SQSTM1	13.8757	0.0194
GO:0001618	virus receptor activity	4	2.7027	ITGB1, LAMP1, RPSA, PVR	6.7276	0.0214
GO:0004488	methylenetetrahydrofolate dehydrogenase (NADP+) activity	2	1.3514	MTHFD1, MTHFD1L	64.7534	0.0303
GO:0019899	enzyme binding	8	5.4054	LAMP1, PCBP2, HMGA1, CAPN2, CTNNB1, SQSTM1, KPNB1, LUC7L2	2.6566	0.0309
GO:0051287	NAD binding	3	2.0270	IDH3B, PHGDH, DLD	9.7130	0.0377
GO:0048487	beta-tubulin binding	3	2.0270	RACGAP1, PEX14, ARL8B	9.2505	0.0412
GO:0019901	protein kinase binding	9	6.0811	RACGAP1, DCTN2, MVP, HSPB1, CTNNB1, AP2A1, VDAC1, PTN, SQSTM1	2.2809	0.0434
**Cellular Component**						
GO:0016020	membrane	66	44.5946	ITGB1, GPI, FEN1, COX4I1, EIF4A3, OLA1, AP2A1, HCCS, PVR, LMNB1, EEA1, SEC61A1, ATIC, APMAP, LAMP1, FLOT1, FLOT2, KPNA2, SEC63, ACTR3, HSDL2, HLA-C, RPSA, MTHFD1, SOAT1, GORASP2, XRN2, GEMIN5, HNRNPH2, VDAC1, RPL26, SLC25A5, ERGIC1, KPNB1, CERS2, ARL8B, FTL, ABCG2, DCTN2, NUFIP2, LAMA4, MVP, DLST, PTN, NTPCR, MTHFD1L, TMEM205, PCBP2, EMC3, EIF4H, PGK1, RPL13, FLNB, ABCF1, CYB5B, SDF2L1, TMEM30A, IMMT, PEX14, SLC4A7, BST2, CTNNB1, EIF3H, HYOU1, RPS24, RPS23	2.4222	<0.0001
GO:0070062	extracellular exosome	49	33.1081	ITGB1, GPI, RNH1, OLA1, HSPB1, EEA1, ALCAM, ATIC, LGALS1, LAMP1, FTH1, CAPN2, FLOT1, FLOT2, CPNE3, PHGDH, ACTR3, CCT2, HLA-C, RPSA, SCAMP3, MTHFD1, THRAP3, TAGLN2, VDAC1, RPL26, SQSTM1, CD44, KPNB1, ARL8B, FTL, DCTN2, LAMA4, MVP, GNS, ACAT2, RACGAP1, DDT, PCBP2, PGK1, FLNB, RDX, SOD1, PPP1CA, CCT6A, BST2, CTNNB1, EIF3H, HYOU1	3.0706	<0.0001
GO:0005925	focal adhesion	19	12.8378	ITGB1, ACTR3, RDX, HMGA1, HSPB1, PVR, MMP14, ALCAM, CTTN, PCBP2, FLOT1, CAPN2, FLOT2, CTNNB1, FLNB, HYOU1, CPNE3, CD44, CD99	6.2165	<0.0001
GO:0005829	cytosol	73	49.3243	GPI, RNH1, COX4I1, RTCB, EIF4A3, OLA1, HSPB1, AP2A1, HCCS, NOC2L, LMNB1, EEA1, ATIC, LGALS1, LAMP1, FTH1, CAPN2, CPNE3, PHGDH, RPL37, UBXN4, KPNA2, ACTR3, EIF5B, CCT2, RPSA, ARMC1, MMP14, MTHFD1, CTTN, GEMIN5, TAGLN2, HNRNPH2, RPL26, DNAJC8, SQSTM1, CD44, KPNB1, ARL8B, FTL, COPB2, SEC23A, DCTN2, NUFIP2, POLDIP3, MVP, DLST, ACAT2, NME1-NME2, MTHFD1L, BAG2, RACGAP1, PCBP2, EIF4H, PGK1, RPL13, FLNB, IGF2BP3, ABCF1, SMARCA5, HMGA1, PEX14,	1.8389	<0.0001

				SOD1, PPP1CA, CCT6A, BST2, MOV10, RPS27, VAPA, CTNNB1, EIF3H, RPS24, RPS23		
GO:0005743	mitochondrial inner membrane	18	12.1622	MRPS17, NDUFB10, UQCRB, MRPS36, NDUFB11, COX4I1, MRPS18B, TIMM13, MRPS18A, IMMT, MRPS21, HCCS, MRPL13, MRPS5, UQCRH, MRPL33, TMEM65, SLC25A5	5.2254	<0.0001
GO:0005739	mitochondrion	31	20.9459	FEN1, NDUFB11, MRPS36, COX4I1, TIMM13, DLST, HCCS, MRPL13, UQCRH, ACAT2, MRPL33, TMEM65, MTHFD1L, IDH3B, CPNE3, DLAT, EIF5B, HSDL2, PDHA1, MTX2, MRPS18B, MRPS18A, IMMT, MRPS5, SOD1, ARMC1, MTHFD1, VDAC1, SLC25A5, DLD, SQSTM1	2.9708	<0.0001
GO:0005840	ribosome	10	6.7568	MRPS17, RPS27, MRPS18B, MRPS18A, RPL13, RPL37, MRPL13, RPS24, RPS23, ABCF1	7.9008	<0.0001
GO:0022626	cytosolic ribosome	7	4.7297	RPS27, RPL13, RPSA, RPL37, RPL26, RPS24, RPS23	12.6413	<0.0001
GO:0044754	autolysosome	4	2.7027	LAMP1, FTH1, SQSTM1, FTL	50.5651	0.0001
GO:0005763	mitochondrial small ribosomal subunit	5	3.3784	MRPS17, MRPS18B, MRPS18A, MRPS21, MRPS5	21.0688	0.0001
GO:0005783	endoplasmic reticulum	21	14.1892	SDF2L1, TMEM30A, ERAP2, WFS1, HLA-C, PTN, SEC61A1, TMX3, CTTN, VAPA, SOAT1, CAPN2, RPL13, HYOU1, UBXN4, SQSTM1, ERGIC1, RPS24, CERS2, RPS23, SEC63	2.5526	0.0002
GO:0005789	endoplasmic reticulum membrane	20	13.5135	COPB2, SEC23A, SDF2L1, ERAP2, TMEM41B, WFS1, RTCB, HLA-C, YIF1A, SEC61A1, SPCS2, TMX3, VAPA, SOAT1, GORASP2, EMC3, UBXN4, KPNA2, ERGIC1, CERS2	2.5145	0.0003
GO:0045254	pyruvate dehydrogenase complex	3	2.0270	PDHA1, DLAT, DLD	69.5270	0.0007
GO:0035577	azurophil granule membrane	5	3.3784	BST2, TMEM30A, VAPA, LAMP1, CPNE3	11.9874	0.0008
GO:0030864	cortical actin cytoskeleton	5	3.3784	CTTN, RDX, FLOT1, CAPN2, FLOT2	11.5878	0.0009
GO:0005967	mitochondrial pyruvate dehydrogenase complex	3	2.0270	PDHA1, DLAT, DLD	52.1453	0.0014
GO:0042788	polysomal ribosome	4	2.7027	NUFIP2, EIF3H, RPS23, ABCF1	16.8550	0.0017
GO:0016600	flotillin complex	3	2.0270	FLOT1, CTNNB1, FLOT2	46.3514	0.0018
GO:0045121	membrane raft	8	5.4054	ITGB1, BST2, FLOT1, CAPN2, PGK1, VDAC1, SLC25A5, ABCG2	4.3970	0.0023
GO:0030027	lamellipodium	7	4.7297	ACTR3, ITGB1, CTTN, RDX, FLOT1, CTNNB1, FLOT2	5.0697	0.0026
GO:0005635	nuclear envelope	7	4.7297	SUN2, RTCB, UBXN4, RAE1, KPNB1, LMNB1, ABCF1	5.0174	0.0027
GO:0022627	cytosolic small ribosomal subunit	4	2.7027	RPS27, RPSA, RPS24, RPS23	12.0917	0.0043
GO:0005912	adherens junction	6	4.0541	RDX, FLOT1, CTNNB1, FLOT2, PVR, PPP1CA	4.7405	0.0088

GO:1990904	ribonucleoprotein complex	6	4.0541	MVP, PCBP2, EIF4A3, HNRNPH2, RPL26, ZC3H14	4.5591	0.0102
GO:1904115	axon cytoplasm	4	2.7027	HSPB1, ZC3H14, ARL8B, SOD1	8.5572	0.0113
GO:1904813	ficolin-1-rich granule lumen	5	3.3784	GPI, MVP, FTH1, GNS, KPNB1	5.6070	0.0121
GO:0045202	synapse	10	6.7568	SLC4A7, EIF5B, RPL13, CTNNB1, VDAC1, RPL37, RPL26, RPS24, RPS23, ARL8B	2.6386	0.0136
GO:0008043	intracellular ferritin complex	2	1.3514	FTH1, FTL	139.0541	0.0142
GO:0015935	small ribosomal subunit	3	2.0270	RPSA, RPS24, RPS23	14.3849	0.0181
GO:0005654	nucleoplasm	40	27.0270	GPI, FEN1, SF3B3, RNH1, NUFIP2, POLDIP3, COX4I1, RTCB, EIF4A3, DLST, NOC2L, LMNB1, RACGAP1, PCBP2, CPNE3, KPNA2, RAE1, ABCF1, SMARCE1, SMARCA5, HMGA1, MRPS18B, RPSA, SOD1, PPP1CA, RPS27, THRAP3, XRN2, GEMIN5, CTNNB1, HNRNPH2, RPL26, DNAJC8, SRSF6, SQSTM1, ERGIC1, RPS24, KPNB1, RPS23, ABCG2	1.4046	0.0187
GO:0101004	cytolytic granule membrane	2	1.3514	LAMP1, ARL8B	92.7027	0.0213
GO:0005788	endoplasmic reticulum lumen	7	4.7297	LGALS1, SDF2L1, IGFBP5, ERAP2, WFS1, COL5A2, HYOU1	3.1810	0.0228
GO:0005758	mitochondrial intermembrane space	4	2.7027	TIMM13, CAPN2, IMMT, SOD1	6.1802	0.0267
GO:0010494	cytoplasmic stress granule	4	2.7027	MOV10, NUFIP2, IGF2BP3, KPNB1	6.1802	0.0267
GO:0005737	cytoplasm	52	35.1351	ITGB1, RNH1, RTCB, EIF4A3, OLA1, HSPB1, PVR, EEA1, LGALS1, LAMP1, MDK, FTH1, CAPN2, CPNE3, RPL37, HMGN2, KPNA2, ACTR3, EIF5B, RPSA, MMP14, CTTN, GEMIN5, RPL26, SQSTM1, KPNB1, ARL8B, FTL, DCTN2, NUFIP2, POLDIP3, MVP, ACAT2, DDT, PCBP2, RPL13, FLNB, IGF2BP3, ZC3H14, RAE1, CD99, ABCF1, ERAP2, MTX2, RDX, SOD1, PPP1CA, CCT6A, BST2, CTNNB1, RPS24, RPS23	1.2928	0.0269
GO:0032839	dendrite cytoplasm	3	2.0270	FLOT2, ZC3H14, SOD1	11.5878	0.0272
GO:0045252	oxoglutarate dehydrogenase complex	2	1.3514	DLST, DLD	69.5270	0.0283
GO:0016323	basolateral plasma membrane	6	4.0541	SLC4A7, FLOT1, CTNNB1, FLOT2, AP2A1, CD44	3.4194	0.0310
GO:0042470	melanosome	4	2.7027	ITGB1, MMP14, LAMP1, FLOT1	5.3482	0.0386
GO:0098685	Schaffer collateral - CA1 synapse	4	2.7027	ITGB1, EEA1, CTNNB1, PTN	5.1502	0.0424
GO:0098978	glutamatergic synapse	8	5.4054	ITGB1, EEA1, EIF4A3, FLOT1, CTNNB1, FLOT2, KPNA2, PPP1CA	2.4503	0.0447

GO:0005757	mitochondrial permeability transition pore complex	2	1.3514	VDAC1, SLC25A5	39.7297	0.0489
